# Individualized prediction models in ADHD: a systematic review and meta-regression

**DOI:** 10.1038/s41380-024-02606-5

**Published:** 2024-05-23

**Authors:** Gonzalo Salazar de Pablo, Raquel Iniesta, Alessio Bellato, Arthur Caye, Maja Dobrosavljevic, Valeria Parlatini, Miguel Garcia-Argibay, Lin Li, Anna Cabras, Mian Haider Ali, Lucinda Archer, Alan J. Meehan, Halima Suleiman, Marco Solmi, Paolo Fusar-Poli, Zheng Chang, Stephen V. Faraone, Henrik Larsson, Samuele Cortese

**Affiliations:** 1 Department of Child and Adolescent Psychiatry, Institute of Psychiatry, Psychology and Neuroscience, King’s College London, London, UK; 2 Child and Adolescent Mental Health Services, South London and Maudsley NHS Foundation Trust, London, UK; 3 Institute of Psychiatry and Mental Health. Department of Child and Adolescent Psychiatry, Hospital General Universitario Gregorio Marañón School of Medicine, Universidad Complutense, Instituto de Investigación Sanitaria Gregorio Marañón (IiSGM), CIBERSAM, Madrid, Spain; 4 Department of Biostatistics and Health Informatics, Institute of Psychiatry, Psychology and Neurosciences, King’s College London, London, UK; 5 King’s Institute for Artificial Intelligence, King’s College London, London, UK; 6 School of Psychology, University of Nottingham, Nottingham, Malaysia; 7 Centre for Innovation in Mental Health—Developmental Lab, School of Psychology, University of Southampton, Southampton, UK; 8 School of Psychology, University of Southampton, Southampton, UK; 9 Post-Graduate Program of Psychiatry, Universidade Federal do Rio Grande do Sul, Porto Alegre, Brazil; 10 National Center for Research and Innovation (CISM), University of São Paulo, São Paulo, Brazil; 11 ADHD Outpatient Program, Hospital de Clínicas de Porto Alegre, Porto Alegre, Brazil; 12 School of Medical Sciences, Faculty of Medicine and Health, Örebro University, Örebro, Sweden; 13 Solent NHS Trust, Southampton, UK; 14 Department of Medical Epidemiology and Biostatistics, Karolinska Institutet, Stockholm, Sweden; 15 Department of Neurology and Psychiatry, University of Rome La Sapienza, Rome, Italy; 16 Institute of Applied Health Research, University of Birmingham, Birmingham, UK; 17 National Institute for Health and Care Research (NIHR), Birmingham Biomedical Research Centre, Birmingham, UK; 18 Department of Psychology, Institute of Psychiatry, Psychology & Neuroscience, King’s College London, London, UK; 19 Yale Child Study Center, Yale School of Medicine, New Haven, CT USA; 20 Departments of Psychiatry and of Neuroscience and Physiology, SUNY Upstate Medical University, Syracuse, Syracuse, NY USA; 21 Department of Psychiatry, University of Ottawa, Ottawa, ON Canada; 22 Department of Mental Health, The Ottawa Hospital, Ottawa, ON Canada; 23 Hospital Research Institute (OHRI) Clinical Epidemiology Program University of Ottawa, Ontario, ON Canada; 24 School of Epidemiology and Public Health, Faculty of Medicine, University of Ottawa, Ottawa, ON Canada; 25 Department of Child and Adolescent Psychiatry, Charité Universitätsmedizin, Berlin, Germany; 26 Early Psychosis: Interventions and Clinical-detection (EPIC) Lab, Department of Psychosis Studies, Institute of Psychiatry, Psychology & Neuroscience, King’s College London, London, UK; 27 Department of Brain and Behavioral Sciences, University of Pavia, Pavia, Italy; 28 Outreach and Support in South-London (OASIS) service, South London and Maudsley NHS Foundation Trust, London, UK; 29 Department of Psychiatry and Psychotherapy, University Hospital, Ludwig-Maximilian-University (LMU), Munich, Germany; 30 Clinical and Experimental Sciences (CNS and Psychiatry), Faculty of Medicine, University of Southampton, Southampton, UK; 31 Hassenfeld Children’s Hospital at NYU Langone, New York University Child Study Center, New York City, NY USA; 32 DiMePRe-J-Department of Precision and Rigenerative Medicine-Jonic Area, University of Bari “Aldo Moro”, Bari, Italy

**Keywords:** ADHD, Biomarkers

## Abstract

There have been increasing efforts to develop prediction models supporting personalised detection, prediction, or treatment of ADHD. We overviewed the current status of prediction science in ADHD by: (1) systematically reviewing and appraising available prediction models; (2) quantitatively assessing factors impacting the performance of published models. We did a PRISMA/CHARMS/TRIPOD-compliant systematic review (PROSPERO: CRD42023387502), searching, until 20/12/2023, studies reporting internally and/or externally validated diagnostic/prognostic/treatment-response prediction models in ADHD. Using meta-regressions, we explored the impact of factors affecting the area under the curve (AUC) of the models. We assessed the study risk of bias with the Prediction Model Risk of Bias Assessment Tool (PROBAST). From 7764 identified records, 100 prediction models were included (88% diagnostic, 5% prognostic, and 7% treatment-response). Of these, 96% and 7% were internally and externally validated, respectively. None was implemented in clinical practice. Only 8% of the models were deemed at low risk of bias; 67% were considered at high risk of bias. Clinical, neuroimaging, and cognitive predictors were used in 35%, 31%, and 27% of the studies, respectively. The performance of ADHD prediction models was increased in those models including, compared to those models not including, clinical predictors (β = 6.54, p = 0.007). Type of validation, age range, type of model, number of predictors, study quality, and other type of predictors did not alter the AUC. Several prediction models have been developed to support the diagnosis of ADHD. However, efforts to predict outcomes or treatment response have been limited, and none of the available models is ready for implementation into clinical practice. The use of clinical predictors, which may be combined with other type of predictors, seems to improve the performance of the models. A new generation of research should address these gaps by conducting high quality, replicable, and externally validated models, followed by implementation research.

## Introduction

Attention-Deficit/Hyperactivity Disorder (ADHD) [1] is a neurodevelopmental condition which is characterized by age-inappropriate and impairing inattention and/or hyperactivity/impulsivity. Over the past decades, neurobiological research has resulted in a shift in the understanding of the pathophysiology of ADHD, from theoretical views of isolated brain dysfunctions to more complex models reflecting the heterogeneity of the clinical manifestations of ADHD [2]. However, neurobiological findings have not yet impacted clinical practice and, currently, the diagnosis of ADHD is exclusively based on a clinical assessment, with no established objective tests being available as standalone tools to diagnose ADHD [3]. The exact factors that predict the persistence of ADHD beyond adolescence are currently unclear. Furthermore, while effective (at least in the short-term) treatments are available [4], there are no established evidence-based prediction models to inform individualized treatment strategies based on the patient’s clinical, environmental, cognitive, genetic, or biological characteristics.

In the last decade, the new field of precision psychiatry has emerged, with the development of multivariable prediction models aimed at predicting the diagnosis, prognosis, or treatment response in relation to several mental health conditions [5, 6], considering individual variability in clinical characteristics, genes, environment, and lifestyle [7]. Advances in the field of prediction modelling have allowed the consolidation of an evidence-based science of precision medicine [8]. Prediction modelling studies investigate the development of such models, as well as their validation [9]. External validity is the extent to which predictions can be generalized to the data from other settings, while internal validity is the extent to which the predictions fit the derivation data [10].

Previous systematic reviews have identified a large number of prediction models across mental health conditions [10, 11]. Notably, in the last few years there has been a rapidly increasing interest in this field, and an emerging number of prediction models on ADHD have been rapidly published, making an updated evaluation of the status of the field essential. Furthermore, to our knowledge, no study has comprehensively and specifically reviewed the status of validated prediction models in ADHD, systematically assessing factors that can affect their predictive performance. Therefore, our primary aim was to systematically review and critically appraise available prediction models that might be considered for clinical use in the identification or management of ADHD. Our secondary aim was to test potential moderating factors that could affect the performance of available models as measured by their area under the curve (AUC), the most reliable and most reported metric across studies.

## Methods

This study (pre-registered protocol: PROSPERO:CRD42023387502) was conducted and reported in accordance with the “Preferred Reporting Items for Systematic Reviews and Meta-analyses” (PRISMA) 2020 and the “Transparent Reporting of a multivariable prediction model for Individual Prognosis Or Diagnosis” (TRIPOD) statements and checklists (Tables S1–4, available online).

### Search strategy and selection criteria

PubMed and Web of Science database including Web of Science Core Collection, BIOSIS Citation Index, KCI-Korean Journal Database, MEDLINE, Russian Science Citation Index, and SciELO Citation Index, Cochrane Central Register of Reviews, Embase and Ovid/PsycINFO databases, were searched from inception until 20/12/2023 with no language restrictions (search terms/syntax in Supplementary 1, available online). The references of the included articles and those in previous relevant reviews were manually searched to identify any possible additional relevant studies. Titles and abstracts were screened, and, after the exclusion of those not relevant, the full texts were assessed against the inclusion and exclusion criteria by a group of researchers who worked independently in pairs on one third of the hits each (GSdP, AB, AC, MD, AC, HS, VP).

The inclusion criteria were: (a) original individual studies; (b) conducted in children and/or adults with ADHD according to established diagnostic criteria (DSM or ICD—any version); (c) reporting on multivariable internally and/or externally [12] validated prediction models; (d) providing diagnostic, prognostic, or treatment-response estimates at the individual subject level or in subgroups; (e) providing at least discrimination as per the AUC (i.e., the ability of the model to separate individuals who develop events from those who do not), accuracy (i.e., the degree of closeness of the measured value), or classification measures (sensitivity, specificity, or predictive values) (definitions in Table 1). The exclusion criteria were: (a) abstracts, conference proceedings, reviews, or meta-analyses; (b) prediction model studies that did not evaluate or report their internal or external validation; (c) predictor-finding studies that included one predictor only.

**Table 1:** Definitions of key terms in prediction science.

Term	Definition
Accuracy	The degree of closeness of the measured value
Area under the curve (AUC)	The area enclosed by the curve of a mathematical function and a reference axis
Calibration	The degree of adjustment of a measurement to account for the sources of variation
Classification	The degree of assignment of an outcome to the correct category
Discrimination	The ability of the model to separate individuals who develop events from those who do not
External validation	Process of evaluating the extent to which the predictions can be generalized to the data from other settings
Internal validation	Process of evaluating the extent to which the predictions fit the derivation data after controlling for overfitting and optimism
Optimism	Increase in the assigned performance values due to methodological bias
Overfitting	A modelling error consisting on a measure being too closely aligned to a previous set of data points
Performance	The degree of execution of a model in regards to discrimination and calibration aspects
Prediction modelling studies	Studies that use statistical procedures to predict the appearance of a condition or outcome.

### Descriptive measures and data extraction

Data extraction items (Supplementary 2, available online) were based on the “Checklist for critical Appraisal and data extraction for systematic Reviews of prediction Modelling Studies” (CHARMS) and the “Transparent Reporting of a multivariable prediction model for Individual Prognosis Or Diagnosis” (TRIPOD) statements. The model’s ability to separate individuals with and without the outcome, e.g., AUC, was selected as the main outcome. Discriminative validity is usually considered ‘acceptable’ when AUC scores are between 0.7–0.8, ‘good’ between 0.8–0.9, and ‘excellent’ when >0.9 [13]. We extracted information on the performance of each model assessed by other measures when reported. When more than one outcome per study was found in the same category, we extracted the information for the primary outcome, as defined in each article, unless the study reported multiple primary co-outcomes. We relied on what the individual authors reported as their primary outcome.

### Quality assessment

Risk of bias was assessed for each of the included studies with a validated version - previously used in mental health research- of the Prediction Model Risk of Bias Assessment Tool (PROBAST v5/05/2019) [9] (Supplementary 3, available online).

### Strategy for data synthesis

Data from the included studies were first summarized in descriptive tables. The top 10% of the most commonly employed predictor types were shown in a bar chart. We then conducted meta-regressions to estimate the association, when data were available, between AUC and: (i) the type of validation (internal vs external); (ii) the age range (children and adolescents vs adults vs combined/not reported); (iii) the type of model (diagnostic vs prognostic vs treatment-response model); (iv) the number of predictors; (v) the type of predictors [clinical/sociodemographic vs any biomarker (neuroimaging, electroencephalography, magnetoencephalography, proteomic, genetic, cognitive, or a combination of modalities)] [10]; (vi) the modality of predictors [unimodal, using only one type of predictor (e.g., clinical only) vs multimodal, using more than one type of predictor (e.g., clinical and biomarker)] and (vii) the quality of the studies (low risk vs unclear risk vs high risk). We used a random-effects model to allow for heterogeneity in underlying associations across studies. Number of studies permitting, we also planned sensitivity analyses to assess the impact of studies being at low risk of bias and without suboptimal validation. Suboptimal validation was appraised by two statisticians (RI and MHI) with a focus on: (1) double dipping (i.e., performing feature selection or selection of tuning -or penalty- parameters on data samples from both the training and the test set) [14]; (2) reporting apparent/non-validated predictive performance instead of the validated predictive performance; (3) reporting the size and significance of apparent regression coefficients rather than the cross-validated performance measure, and (4) re-estimating regression coefficients in the test set, instead of applying the apparently validated model. The meta-regression was performed with Comprehensive Meta-Analysis Version 3. Statistical significance was set at p < 0.05.

## Results

**Fig. 1:**
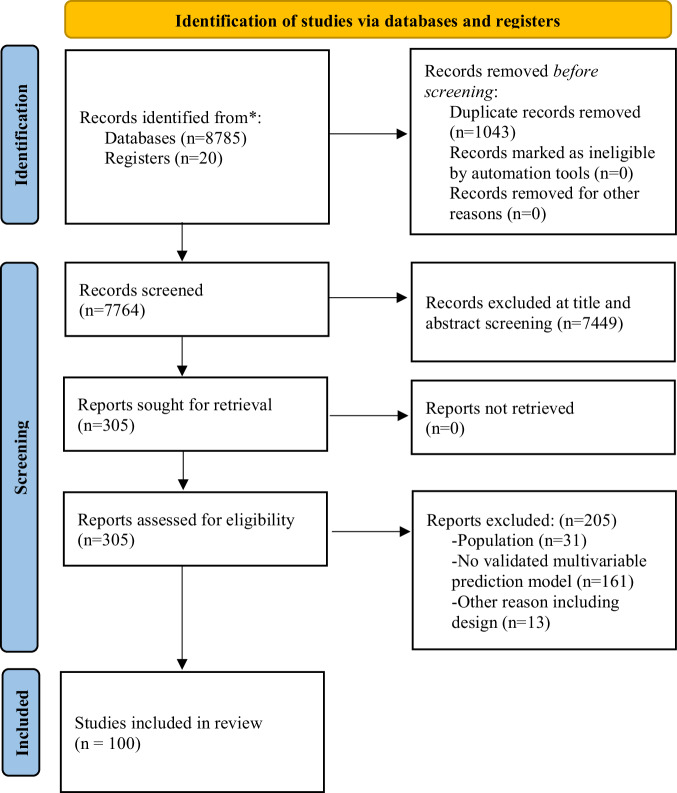
PRISMA flowchart. Preferred reporting items for systematic reviews and meta-analyses (PRISMA) flowchart outlining study selection process.

**Fig. 2:**
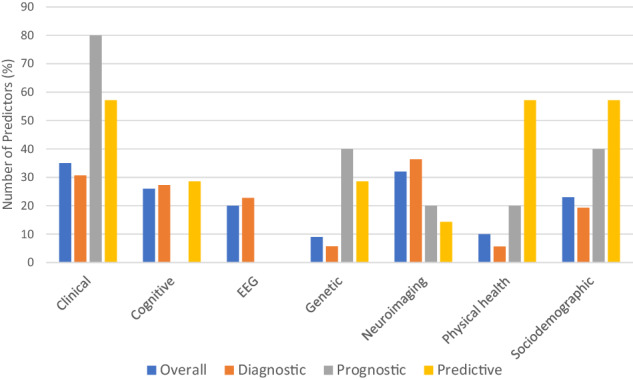
Reported predictors. Most frequently reported predictors across prediction model types.

After removing duplicates, from an initial pool of 7764 references, we retained 100 eligible studies (Fig. 1). None of the models reported in the included studies was implemented into clinical practice. 96 (96.0%) and seven (7.0%) models were internally and externally validated, respectively. Among the eligible studies, 88.0% reported on diagnostic prediction models, 5.0% on prognostic models (with outcomes such as symptom change or development of substance use disorders), and 7.0% on treatment-response models. The retained studies most frequently used clinical (35.0%), neuroimaging (31.0%) and cognitive (27.0%), predictors (Fig. 2). The total sample size was 323,554 individuals, ranging from 10 to 238,696 individuals per study. The average age was 15.7 years. The source of data encompassed case-control studies (73 studies, 73.0%), cohort studies (23 studies, 23.0%), and clinical trials (4 studies, 4.0%). AUC was the most commonly reported measure of model performance (61.0%), followed by accuracy (36.0%). Eight studies (8.0%) only reported the sensitivity and specificity of the models.

### Predictors in prediction models

In the 88 diagnostic prediction models, studies used cognitive (K = 5 studies) [15–19], clinical (K = 13) [20–31]^287^, neuroimaging (K = 19) [32–50], EEG (K = 15) [51–65], genetic (K = 2) [66, 67], ECG (K = 2) [68, 69], physical health (K = 1) [70], EEG and cognitive (K = 4) [71–74], sociodemographic and neuroimaging (K = 4) [75–77]^294^, clinical and cognitive (K = 4) [78–81], sociodemographic and cognitive (K = 2) [82, 83], cognitive and physical health (K = 2) [84, 85], genetic and neuroimaging (K = 2) [86, 87], sociodemographic and genetic (K = 1) [88], clinical and sociodemographic (K = 1) [89] EEG and EMG (K = 1) [90], sociodemographic and neuroimaging (K = 2) [91, 92], sociodemographic, clinical and cognitive (K = 3) [93–95], cognitive, sociodemographic and neuroimaging (K = 1) [96], clinical, sociodemographic and neuroimaging (k = 1) [97], clinical, cognitive and neuroimaging (k = 1) [98] and sociodemographic, clinical, cognitive and physical health (K = 2) [99, 100] predictors.

In the 5 prognostic prediction models, studies employed sociodemographic and clinical (K = 2) [101, 102], physical health and clinical (K = 1) [103], neuroimaging and genetic (K = 1) [104], and clinical and genetic (K = 1) [105] predictors.

In the 7 treatment-response prediction models, studies relied on neuroimaging (K = 1) [106], genetic (k = 1) [107], sociodemographic, clinical, cognitive, and physical health (K = 1) [108], genetic, cognitive and physical health (K = 1) [109], clinical, sociodemographic, service use and physical health (K = 1) [110], sociodemographic and clinical predictors (K = 1) [111], and sociodemographic, clinical and physical health (K = 1) predictors [112].

### Performance of prediction models

The performance of ADHD prediction models was highly variable, with AUC ranging from 0.50 to 0.99. AUC ranged from 0.50 to 0.96 in diagnostic models, from 0.73 to 0.87 in prognostic models, and from 0.72 to 0.99 in models for predicting treatment-response. Accuracy ranged from 0.53 to 1.0 (0.53 to 1.0 for diagnostic models, 0.73 to 0.87 for prognostic models, and 0.72 to 0.88 for treatment-response models) (Tables S5–7, available online). Model calibration was assessed in 6.0% of the studies.

### Meta-regression results

The performance of ADHD prediction models was increased in those models including (K = 26), as compared to those not including, clinical predictors (K = 36) (β = 6.540, p = 0.007). No significant findings emerged when considering type of validation (internal K = 58 vs external K = 4), age range (children and adolescents K = 33 vs adults K = 11 vs combined/not reported K = 18), type of prediction model (diagnostic K = 52 vs prognostic K = 3 vs treatment-response K = 7) (p > 0.05), number of predictors (K = 34), other types of predictors comparisons (clinical/service use/sociodemographic K = 13 vs biomarkers K = 33 vs combination K = 17) or quality of the studies (low risk K = 7 vs unclear risk K = 11 vs high risk K = 44) (all p > 0.05) (Table 2), according to our meta-regression analyses.

**Table 2:** Meta-regressions exploring the possible moderating factors impacting the area under the curve (AUC).

Moderating factors	Number of studies	Meta-regression Coefficient	SE	Z value	P	95%CI
(i) Type of validation						
External vs Internal validation	62 (58;4)	2.319	–3.79	0.75	0.454	–3.790; 8.474
(ii) Age range						
Children&adolescents vs adults	44 (33;11)	–1.612	2.976	–0.54	0.588	–7.444; 4.221
Children&adolescents vs combined/not reported	51 (33;18)	0.482	2.696	0.18	0.858	–4.802; 5.766
Adults vs combined/not reported	29 (11;18)	2.093	3.344	0.63	0.531	–4.460; 8.647
(iii) Type of model						
Diagnostic vs prognostic	55 (52;3)	–2.073	5.786	–0.36	0.720	–13.413; 9.268
Diagnostic vs treatment-response	59 (52;7)	1.045	3.538	0.30	0.7677	–5.889; 7.979
Prognostic vs treatment-response	10 (3;7)	3.118	6.514	0.48	0.632	–9.649; 15.885
(iv) Number of predictors						
Number of predictors	34	–0.0047	0.003	–1.39	0.166	–0.011; 0.002
(v) Type of predictors						
Clinical or service use or sociodemographic vs biomarkers	46 (13; 33)	–1.249	4.337	–0.29	0.773	–9.750; 7.252
Clinical or service use or sociodemographic vs combination	30 (13;17)	–2.749	2.935	–0.94	0.349	–8.502; 3.003
Biomarkers vs combination	50 (33;17)	–0.404	3.786	–0.11	0.915	–7.825; 7.017
Including clinical vs not including clinical	62 (26;36)	–6.540	2.410	–2.72	**0.007**	–11.264; –1.824
(vi) Modality of predictors						
Unimodal vs Multimodal	62 (36;26)	–3.496	1.873	–1.87	0.062	–7.167; 0.174
(vii) Quality assessment						
Low risk vs unclear risk	18 (7;11)	3.629	5.058	0.72	0.473	–6.284; 13.542
Low risk vs high risk	51 (7; 44)	5.549	3.910	1.42	0.156	–6.284; 13.542
Unclear risk vs high risk	55 (11; 44)	1.920	3.999	0.48	0.631	–5.918; 9.759

### Quality of prediction models

**Fig. 3:**
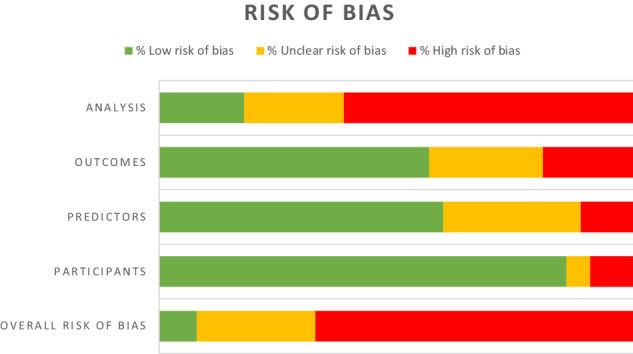
Quality assessment. Risk of bias of the retrieved studies, as assessed by the PROBAST tool.

Sixty-seven (67.0%) of the included studies were deemed to be at high risk of bias according to the PROBAST tool. The results from the different domains were heterogeneous: 9 (9.0%) were at high risk of bias in the participants domain, 11 (11.0%) in the predictors domain, 19 (19.0%) in the outcomes domain, and 61 (61.0%) in the analysis domain. Only 8 (8.0%) of the included studies (seven diagnostic and one prognostic) were considered to be at overall low risk of bias; 86 (86.0%) of the studies were deemed at low risk of bias in the participants domain, 60 (60.0%) in the predictors domain, 57 (57.0%) in the outcomes domain, and 18 (18.0%) in the analysis domain (Table S8, available online; Fig. 3). In 13 studies (13.0%) the performance was evaluated in development dataset only, resulting in suboptimal validation (Table S9, available online).

## Discussion

This is the first systematic review to quantitatively summarize the evidence regarding internally or externally validated diagnostic, prognostic, or treatment-response prediction models specifically in the field of ADHD, appraising the quality of the models and assessing possible factors affecting their performance in terms of AUC. Among the 100 prediction modelling studies included, 88% reported on diagnostic, 5% on prognostic, and 7% on treatment-response models. Furthermore, 35% of studies used clinical, 31.0% neuroimaging, and 27.0% cognitive predictors. Notably, only 7.0% of models were externally validated. The performance of ADHD prediction models was increased in those models including, compared to those models not including, clinical predictors. Meta-regressions did not detect any significant changes in the AUC according to other evaluated variables. Also, 67.0% of included studies were found to be at high risk of bias according to PROBAST quality assessment.

Our review shows that the number of prediction models in the field of ADHD is increasing exponentially over the years, with a wide range of predictors that might potentially support the diagnosis of ADHD, and, to a lesser extent, the understanding of the clinical progression of the disorder or the factor influencing the response to interventions.

However, the discrimination and accuracy of the models, although good, may not be enough for implementation into clinical practice. This emerging body of research is limited by not only a small number of externally validated models, but also, and crucially, by lack of implementation research in real-world clinical practice. Our findings align with previous evidence related to other mental health conditions suggesting that external validation of prediction models is still infrequent in psychiatry/mental health [113]. A similar review exploring prediction models across any mental health condition found that only 20.1% of all prediction models were externally validated [11]. Another review found that 30.3% of all models were externally validated following strict validation criteria (4.6% of the total models) [10]. This is in contrast with the status of prediction science in other areas of medicine. For instance, several models have been externally validated between five to seventeen times in the field of chronic obstructive pulmonary disease [114]. Similar approaches may move the field of ADHD forward, ensuring generalizability of the model to clinical populations not used to develop the model.

Within the internally and externally validated models, there was no significant correlation seen between the internal and external performance measures. However, the number of models internally and externally validated (six studies) was low. Our findings may also reflect a suboptimal quality during the internal validation of the models, potentially leading to optimism in the reported performance measures and high risk of bias. In fact, 67.0% of the included studies were found to be at high risk of bias. The “analysis” domain, where 61.0% of the included studies were found to be at high risk of bias seems particularly problematic. Also, calibration was assessed in 6.0% of the studies only. Future prediction models need to make sure that: (1) the sample size is appropriate and there is an appropriate number of participants developing the outcome (which may vary depending on the population and outcome of interest); (2) the number of predictors is appropriate, (3) the missing data is handled appropriately (of note, >80% studies did not report how they handled missing data, 7.0% deleted missing data and only 4.0% carried out multiple imputation techniques, being low adherence to ADHD treatment frequent); (4) complexities in the data (e.g. competing risks, sampling of controls) are accounted for appropriately, and (5) model overfitting and optimism in model performance are accounted for, among other key criteria to develop and validate prediction models [9]. We note that risk of bias was heterogeneous across the different PROBAST domains: only 9.0% of models were at high risk of bias in the participants domain and only 11.0% were at high risk of bias in the predictors domain. Given the strictness of PROBAST scoring thresholds, this highlights some strengths among published ADHD prediction models in the selection of participants and predictors.

In terms of the aim of the models, most of them were intended to support the prediction of ADHD diagnosis (88/100), reflecting an increasing interest in developing diagnostic prediction models in the ADHD field, alongside other mental health conditions such as depression [115], first episode psychosis [116], or bipolar disorder [117], following a similar route, likely due to the perception of the suboptimal nature of a “subjective” diagnosis. Notably, unlike other mental health conditions, where performance in diagnostic models has been found to be superior to that in prognostic and treatment-response models [10], we did not find this to be the case in the field of ADHD. The limited number of available treatment-response models points to a critical need for carefully designed experimentally controlled trials (or high quality observational studies) to identify biomarkers that index inter-individual variability and predict treatment response [118]. While studies on treatment-response models are complex to perform, mostly due to the intervention-related components (particularly randomized clinical trials), as well as to ethical issues [10], observational studies relying on electronic health-care records on the long-term effectiveness and safety of the interventions could provide a meaningful alternative [119].

In terms of predictor types, a significant proportion of the reviewed prediction models included clinical predictors, followed closely by neuroimaging predictors and cognitive predictors. The performance of ADHD prediction models was higher in those models including clinical predictors, compared to those models not including clinical predictors. Thus, the use of clinical predictors, which may be combined with other type of predictors, may improve the performance of the models and their inclusion should be considered in prediction models [81]. However, it is important to note that further research is needed to validate these results across different populations, and including additional predictors [99]. While clinical predictors seem to be clearly predominant in other fields [10], in the ADHD field different biomarkers have commonly been used to aid the detection and correct characterization of ADHD. However, there is currently no biomarker in any neurodevelopmental condition, including ADHD, for which there is evidence from two or more studies from independent research groups, with results going into the same direction and of specificity and sensitivity of at least 80% [3]. This makes it difficult to recommend the use of any specific individual predictor in isolation, for future prediction models. Notably, we also found no evidence that multimodal prediction models achieved higher accuracy than unimodal models, arguing against the development of complex models with a wide variety of biomarkers and predictors (which would also be more difficult to apply and implement). In other words, we found no evidence that more complex prediction models encompassing biomarkers or a large number of predictors (which may be more prone to overfitting issues) outperformed less complex models. However, from a quality perspective, five of the six studies assessed at low risk of bias were multimodal, so caution is recommended in the interpretation of this finding.

Future studies should consider net benefit approaches for the evaluation of prediction models for ADHD, which were not used in any of the studies in this review. Net benefit approaches put the benefits and harms of using a prediction model on the same scale, to allow assessment of the relative value associated with using prediction models to guide clinical decision making, over other patient management strategies [120] an approach which is currently lacking in the ADHD prediction literature. 74% of the studies were case-control studies which tried to differentiate individuals with ADHD and healthy controls. Future studies should also try to differentiate ADHD from other relevant syndromes such as the cognitive disengagement syndrome (CDS) -or sluggish cognitive tempo-. CDS is an emerging condition -as opposed to a transdiagnostic phenomenon- in the field of child, adolescent and adult psychiatry [121, 122]. The presence of CDS is particularly important as misdiagnosis of this condition may result in a poor response to first-line treatment with methylphenidate and unwanted side effects [123]. Furthermore, among children crossing into adolescence with ADHD, CDS can result in poor physical activity and behaviour [122].

Our study should be considered in the light of its limitations. Our study has several limitations that must be taken into consideration, mainly related to issues in the available studies rather than in our methods. The main limitation rests in the heterogeneity of the characteristics of prediction models developed in the included studies. The predictors used to develop the models varied considerably across studies. Therefore, in line with previous studies [10], we did not attempt to meta-analyse the categories of prediction models; rather, we presented only meta-regression analyses, stratifying the models for methodological features. We also could not conduct meta-regressions on the studies at low risk of bias and without suboptimal methodological strategies in regard to validation. The sample size and the quality of the studies was highly heterogeneous, with high risk of bias observed in 67.0% of included studies according to the PROBAST criteria, including 61.0% in the analysis domain. Final scores of the PROBAST should be taken with caution as the thresholds are stringent and an outcome is considered to be at high risk of bias when one or more of the questions is answered as not appropriate. We did not analyse the differences among validation measures, some of them being prone to data leakage and inflated accuracy or overfitting. We might have missed relevant studies, particularly if not published. Finally, we could not provide data about calibration as this was rarely reported.

In conclusion, several validated prediction models have been proposed to support the diagnosis of ADHD. However, efforts to predict prognostic outcomes or treatment response to ADHD have been limited. Advances in the field are limited by lack of implementation research in real-world clinical practice. A new generation of research should address these gaps by conducting high quality, replicable and externally validated models. Once an evidence-based model is available, efforts to disseminate it and implement it into clinical practice are recommended.

## Supplementary information

Supplementary material
