# Supplementary material for: Individualized prediction models in ADHD: a systematic review and meta-regression
Source: Mol Psychiatry. 2024 May 23;29(12):3865–73. doi: 10.1038/s41380-024-02606-5 (PMC11609101; doi:10.1038/s41380-024-02606-5)
Supplement: Supplementary file 1 — Supplementary material [file 41380_2024_2606_MOESM1_ESM.docx]

**SUPPLEMENTARY MATERIAL**

**Table S1, p2-5:** PRISMA 2020 statement and checklist

**Table S2, p6:** PRISMA 2020 abstract checklist

**Table S3, p7-9:** TRIPOD statement and checklist

**Table S4, p10-11**: TRIPOD abstract checklist

**Table S5, p12-14**: Reasons for exclusion

**Table S6, p15-20:** Characteristics of diagnostic risk estimation models

**Table S7, p21:** Characteristics of prognostic risk estimation models

**Table S8, p22:** Characteristics of predictive risk estimation models

**Table S9, p23-24:** Quality assessment results

**Table S10, p25-26:** Statistical evaluation of validation features.

**Supplement 1, p27:** Literature search

**Supplement 2, p28:** Variables included in the study

**Supplement 3, p29:** Quality assessment and risk of bias items (PROBAST v5/05/2019)

**This supplementary material has been provided by the authors to give readers additional information about their work.**

**Table S1: PRISMA 2020 statement and checklist**^1^**.**

| **Section and Topic** | **Item #** | **Checklist item** | **Location where item is reported** |
| --- | --- | --- | --- |
| **TITLE** | | |  |
| Title | 1 | Identify the report as a systematic review. | Title page |
| **ABSTRACT** | | |  |
| Abstract | 2 | See the PRISMA 2020 for Abstracts checklist. | Abstract page |
| **INTRODUCTION** | | |  |
| Rationale | 3 | Describe the rationale for the review in the context of existing knowledge. | Introduction |
| Objectives | 4 | Provide an explicit statement of the objective(s) or question(s) the review addresses. | Introduction |
| **METHODS** | | |  |
| Eligibility criteria | 5 | Specify the inclusion and exclusion criteria for the review and how studies were grouped for the syntheses. | Methods |
| Information sources | 6 | Specify all databases, registers, websites, organisations, reference lists and other sources searched or consulted to identify studies. Specify the date when each source was last searched or consulted. | Methods |
| Search strategy | 7 | Present the full search strategies for all databases, registers and websites, including any filters and limits used. | eMethods1 |
| Selection process | 8 | Specify the methods used to decide whether a study met the inclusion criteria of the review, including how many reviewers screened each record and each report retrieved, whether they worked independently, and if applicable, details of automation tools used in the process. | Methods |
| Data collection process | 9 | Specify the methods used to collect data from reports, including how many reviewers collected data from each report, whether they worked independently, any processes for obtaining or confirming data from study investigators, and if applicable, details of automation tools used in the process. | Methods |
| Data items | 10a | List and define all outcomes for which data were sought. Specify whether all results that were compatible with each outcome domain in each study were sought (e.g. for all measures, time points, analyses), and if not, the methods used to decide which results to collect. | eMethods2 |
|  | 10b | List and define all other variables for which data were sought (e.g. participant and intervention characteristics, funding sources). Describe any assumptions made about any missing or unclear information. | eMethods2 |
| Study risk of bias assessment | 11 | Specify the methods used to assess risk of bias in the included studies, including details of the tool(s) used, how many reviewers assessed each study and whether they worked independently, and if applicable, details of automation tools used in the process. | eMethods3 |
| Effect measures | 12 | Specify for each outcome the effect measure(s) (e.g. risk ratio, mean difference) used in the synthesis or presentation of results. | Methods |
| Synthesis methods | 13a | Describe the processes used to decide which studies were eligible for each synthesis (e.g. tabulating the study intervention characteristics and comparing against the planned groups for each synthesis (item #5)). | Methods |
|  | 13b | Describe any methods required to prepare the data for presentation or synthesis, such as handling of missing summary statistics, or data conversions. | Methods |
|  | 13c | Describe any methods used to tabulate or visually display results of individual studies and syntheses. | Methods |
|  | 13d | Describe any methods used to synthesize results and provide a rationale for the choice(s). If meta-analysis was performed, describe the model(s), method(s) to identify the presence and extent of statistical heterogeneity, and software package(s) used. | Methods |
|  | 13e | Describe any methods used to explore possible causes of heterogeneity among study results (e.g. subgroup analysis, meta-regression). | Methods |
|  | 13f | Describe any sensitivity analyses conducted to assess robustness of the synthesized results. | Methods |
| Reporting bias assessment | 14 | Describe any methods used to assess risk of bias due to missing results in a synthesis (arising from reporting biases). | eMethods3 |
| Certainty assessment | 15 | Describe any methods used to assess certainty (or confidence) in the body of evidence for an outcome. | Methods |
| **RESULTS** | | |  |
| Study selection | 16a | Describe the results of the search and selection process, from the number of records identified in the search to the number of studies included in the review, ideally using a flow diagram. | Results, figure 1 |
|  | 16b | Cite studies that might appear to meet the inclusion criteria, but which were excluded, and explain why they were excluded. | NA |
| Study characteristics | 17 | Cite each included study and present its characteristics. | eTables5-7 |
| Risk of bias in studies | 18 | Present assessments of risk of bias for each included study. | eTable8 |
| Results of individual studies | 19 | For all outcomes, present, for each study: (a) summary statistics for each group (where appropriate) and (b) an effect estimate and its precision (e.g. confidence/credible interval), ideally using structured tables or plots. | Results, tables |
| Results of syntheses | 20a | For each synthesis, briefly summarise the characteristics and risk of bias among contributing studies. | Results |
|  | 20b | Present results of all statistical syntheses conducted. If meta-analysis was done, present for each the summary estimate and its precision (e.g. confidence/credible interval) and measures of statistical heterogeneity. If comparing groups, describe the direction of the effect. | Results, Table 2 |
|  | 20c | Present results of all investigations of possible causes of heterogeneity among study results. | Results |
|  | 20d | Present results of all sensitivity analyses conducted to assess the robustness of the synthesized results. | Results, Table 2 |
| Reporting biases | 21 | Present assessments of risk of bias due to missing results (arising from reporting biases) for each synthesis assessed. | Results |
| Certainty of evidence | 22 | Present assessments of certainty (or confidence) in the body of evidence for each outcome assessed. | Results |
| **DISCUSSION** | | |  |
| Discussion | 23a | Provide a general interpretation of the results in the context of other evidence. | Discussion |
|  | 23b | Discuss any limitations of the evidence included in the review. | Discussion, |
|  | 23c | Discuss any limitations of the review processes used. | Discussion |
|  | 23d | Discuss implications of the results for practice, policy, and future research. | Discussion |
| **OTHER INFORMATION** | | |  |
| Registration and protocol | 24a | Provide registration information for the review, including register name and registration number, or state that the review was not registered. | Methods |
|  | 24b | Indicate where the review protocol can be accessed, or state that a protocol was not prepared. | Methods |
|  | 24c | Describe and explain any amendments to information provided at registration or in the protocol. | NA |
| Support | 25 | Describe sources of financial or non-financial support for the review, and the role of the funders or sponsors in the review. | Discussion |
| Competing interests | 26 | Declare any competing interests of review authors. | Discussion |
| Availability of data, code and other materials | 27 | Report which of the following are publicly available and where they can be found: template data collection forms; data extracted from included studies; data used for all analyses; analytic code; any other materials used in the review. | Discussion |

**Table S2:** **PRISMA 2020 abstract checklist.**

| **Section and Topic** | **Item #** | **Checklist item** | **Reported (Yes/No)** |
| --- | --- | --- | --- |
| **TITLE** | | |  |
| Title | 1 | Identify the report as a systematic review. | Yes |
| **BACKGROUND** | | |  |
| Objectives | 2 | Provide an explicit statement of the main objective(s) or question(s) the review addresses. | Yes |
| **METHODS** | | |  |
| Eligibility criteria | 3 | Specify the inclusion and exclusion criteria for the review. | Yes |
| Information sources | 4 | Specify the information sources (e.g. databases, registers) used to identify studies and the date when each was last searched. | Yes |
| Risk of bias | 5 | Specify the methods used to assess risk of bias in the included studies. | Yes |
| Synthesis of results | 6 | Specify the methods used to present and synthesise results. | Yes |
| **RESULTS** | | |  |
| Included studies | 7 | Give the total number of included studies and participants and summarise relevant characteristics of studies. | Yes |
| Synthesis of results | 8 | Present results for main outcomes, preferably indicating the number of included studies and participants for each. If meta-analysis was done, report the summary estimate and confidence/credible interval. If comparing groups, indicate the direction of the effect (i.e. which group is favoured). | Yes |
| **DISCUSSION** | | |  |
| Limitations of evidence | 9 | Provide a brief summary of the limitations of the evidence included in the review (e.g. study risk of bias, inconsistency and imprecision). | Yes |
| Interpretation | 10 | Provide a general interpretation of the results and important implications. | Yes |
| **OTHER** | | |  |
| Funding | 11 | Specify the primary source of funding for the review. | No |
| Registration | 12 | Provide the register name and registration number. | Yes |

**Table S3: TRIPOD statement and checklist**^2^**.**

| **Section and Topic** | **Item No** | **Checklist item** | **Location where item is reported** |
| --- | --- | --- | --- |
| **TITLE** | | |  |
| Title | 1 | Identify the report as a systematic review or meta-analysis (or both) of diagnostic or prognostic model studies. Specify the target population and outcome(s) predicted as relevant to the review question. | Title page |
| **ABSTRACT** | | |  |
| Abstract | 2 | See the TRIPOD-SRMA for Abstracts checklist. | Abstract page |
| **INTRODUCTION** | | |  |
| Rationale | 3 | Describe the rationale for the review in the context of existing knowledge. | Introduction |
| Objectives | 4 | Provide an explicit statement of the objective(s) being addressed with reference to: target population, index and comparator models (as relevant), outcome(s), time (prediction horizon and intended moment of using the model), and setting. | Introduction |
| **METHODS** | | |  |
| Eligibility criteria | 5 | Specify study characteristics used as eligibility criteria, including any prediction models of specific interest, and whether development or validation studies (or both) were eligible. | Methods |
| Information sources | 6 | Specify all databases, registers, websites, organisations, reference lists and other sources searched or consulted to identify studies. Specify the date when each source was last searched or consulted. | Methods |
| Search strategy | 7 | Present the full search strategies for all databases, registers and websites, including any filters and limits used. | eMethods1 |
| Selection process | 8 | Specify the methods used to decide whether a study met the inclusion criteria of the review, including how many reviewers screened each record and each report retrieved, whether they worked independently, and if applicable, details of automation tools used in the process. | Methods |
| Data collection process | 9 | Specify the methods used to collect data from study reports, including how many reviewers collected data from each report, whether they worked independently, any processes for obtaining or confirming data from study investigators, and if applicable, details of automation tools used in the process. | Methods |
| Data items | 10a | List and define all outcomes for which data were sought from each study. | eMethods 2 |
|  | 10b | State the model performance measures that were sought (eg, measures of calibration, discrimination, overall model fit, clinical utility). | Methods |
|  | 10c | Describe how any desired but unreported data items (items 10a, 10b) were handled (eg, contacted authors, calculated from other reported information). | Methods |
| Risk of bias and applicability  assessment | 11 | Specify the methods used to assess risk of bias in the included studies and their applicability to the review question. This should be done separately for each model development and validation. Include details of any tool(s) used, how many reviewers assessed each study and whether they worked independently. | Methods, |
| Synthesis methods | 12a | Describe any methods for synthesising estimates of performance measures for each model. If meta-analysis was carried out, describe the methods used, including any transformations of data before pooling, how any heterogeneity in model performance was quantified and handled, and software package(s) used. | Methods |
|  | 12b | Describe any methods used to explore possible causes of heterogeneity in model performance (eg, subgroup analysis, metaregression), including whether or not they were planned.. | Methods |
|  | 12c | Describe any sensitivity analyses conducted to assess robustness of the synthesised results. | N.a. |
| Certainty assessment | 13 | Describe any methods used to assess certainty (or confidence) in the body of evidence for a prediction model. | Methods |
| **RESULTS** | | |  |
| Study selection | 14 | Describe the results of the search and selection process, from the number of records identified in the search to the number of studies and models included in the review, ideally using a flow diagram. | Results, figure 1 |
| Study and model characteristics | 15 | Present study characteristics and model details extracted (as per item 10a), and cite the study reports. | Tables S5-7 |
| Risk of bias and applicability | 16 | Present results of risk of bias and applicability assessment. | Table S8 |
| Results of model performance | 17 | Present performance estimates for each model and all evaluations. | Results, tables |
| Results of syntheses | 18a | Present the results of any synthesis of model performance, together with details of which study estimates contributed. If metaanalysis was carried out, then for each model and performance measure, present summary results, confidence/credible intervals,  and measures of heterogeneity. Forest plots may be useful. | Results |
|  | 18b | For each model, present results of all investigations of possible causes of heterogeneity in model performance. | N.a. |
|  | 18c | Present results of all sensitivity analyses conducted to assess the robustness of the synthesised results. | N.a. |
| Certainty of evidence | 22 | Present assessments of certainty (or confidence) in the body of evidence for each outcome assessed. | Results |
| **DISCUSSION** | | |  |
| Summary of evidence | 20 | Summarise the main findings including the strengths and limitations of the evidence. | Discussion |
| Limitations | 21 | Discuss the strengths and limitations of the review process. | Discussion |
| Implications | 22 | Discuss implications of the results in the context of other evidence and for practice, policy, and future research. | Discussion |
| **OTHER INFORMATION** | | |  |
| Registration and protocol | 23a | Provide registration information for the review, including register name and registration number, or state that the review was not registered. | Methods |
|  | 23b | Indicate where the review protocol can be accessed, or state that a protocol was not prepared. | Methods |
|  | 23c | Describe and explain any amendments to information provided at registration or in the protocol. | Methods |
| Support | 24 | Describe sources of financial or non-financial support for the review, and the role of the funders or sponsors in the review. | Discussion |
| Competing interests | 25 | Declare any competing interests of review authors. | Discussion |
| Availability of data, code and other materials | 26 | Report which of the following are publicly available and where they can be found: template data collection forms; data extracted from included studies; data used for all analyses; analytic code; any other materials used in the review. | Discussion |

**Table S4:** **TRIPOD statement abstract checklist.**

| **Section and Topic** | **Item #** | **Checklist item** | **Reported (Yes/No)** |
| --- | --- | --- | --- |
| **TITLE** | | |  |
| Title | 1 | Identify the report as a systematic review or meta-analysis (or both) of diagnostic or prognostic model studies. Specify the target population and outcome(s) predicted as relevant to the review question. | Yes |
| **BACKGROUND** | | |  |
| Objectives | 2 | Provide an explicit statement of the main objective(s) being addressed with reference to: target population, index and comparator models (as relevant), outcome(s), time (prediction horizon and intended moment of using the model), and setting | Yes |
| **METHODS** | | |  |
| Eligibility criteria | 3 | Specify study characteristics used as eligibility criteria, including any prediction models of specific interest, and whether development or validation studies (or both) were eligible | Yes |
| Information sources | 4 | Specify the information sources (e.g. databases, registers) used to identify studies and the date when each was last searched. | Yes |
| Risk of bias | 5 | Specify the methods used to assess risk of bias in the included studies. | Yes |
| Synthesis of results | 6 | Specify the methods used to synthesise performance measures for each model of interest. | Yes |
| **RESULTS** | | |  |
| Included studies | 7 | Give the total number of included studies and models,and summarise relevant study characteristics and model details. | Yes |
| Synthesis of results | 8 | Present results for each of the main models of interest. If meta-analysis was used to synthesise study estimates of model performance, report the summary result and confidence/credible interval for each performance measure, together with the number of study estimates contributing. | Yes |
| **DISCUSSION** | | |  |
| Limitations of evidence | 9 | Provide a brief summary of the limitations of the evidence included in the review. | Yes |
| Interpretation | 10 | Provide a general interpretation of the results and important implications for research and practice. | Yes |
| **OTHER** | | |  |
| Funding | 11 | Specify the primary source of funding for the review. | No |
| Registration | 12 | Provide the register name and registration number. | Yes |

**Table S5 Reasons for exclusion**

| **Article** | **Reason for exclusion** | **Article** | **Reason for exclusion** |
| --- | --- | --- | --- |
| Abello 2022^3^ | No validated multivariable prediction model | Johnston 2014^4^ | No validated multivariable prediction model |
| Aceves-Fernandez 2021^5^ | No validated multivariable prediction model | Joshi 2021^6^ | No validated multivariable prediction model |
| Agarwal 2022^7^ | Population | Karam 2015^8^ | No validated multivariable prediction model |
| Ahmadi 2021^9^ | No validated multivariable prediction model | Kaur 2018^10^ | No validated multivariable prediction model |
| Ahmadlou 2010^11^ | No validated multivariable prediction model | Kaur 2022^12^ | Other reason including design |
| Amado-Caballero 2020^13^ | No validated multivariable prediction model | Kessler 2005^14^ | No validated multivariable prediction model |
| Alameda 2021^15^ | No validated multivariable prediction model | Khoshnoud 2015^16^ | No validated multivariable prediction model |
| An 2018^17^ | Population | Kiiski 2020^18^ | No validated multivariable prediction model |
| Anderson 2014^19^ | No validated multivariable prediction model | Kiiski 2020^20^ | No validated multivariable prediction model |
| Ardulov 2021^21^ | Other reason including design | Kim 2020^22^ | No validated multivariable prediction model |
| Areces 2020^23^ | No validated multivariable prediction model | Kooij 2013^24^ | No validated multivariable prediction model |
| Aydin 2022^25^ | No validated multivariable prediction model | Kuang 2014^26^ | No validated multivariable prediction model |
| Balboni 2017^27^ | No validated multivariable prediction model | Kushki 2019^28^ | No validated multivariable prediction model |
| Basaran 2020^29^ | Other reason including design | Kurokami 2022^30^ | No validated multivariable prediction model |
| Bashiri 2018^31^ | No validated multivariable prediction model | Labarga 2019^32^ | No validated multivariable prediction model |
| Bedolla-Ibarra 2022^33^ | No validated multivariable prediction model | Lan 2021^34^ | No validated multivariable prediction model |
| Bell 2022^35^ | No validated multivariable prediction model | Lancaster 2018^36^ | Population |
| Bener 2013^37^ | No validated multivariable prediction model | Lanka 2020^38^ | Population |
| Bener 2014^39^ | No validated multivariable prediction model | Lanka 2020^40^ | No validated multivariable prediction model |
| Bennett 2009^41^ | No validated multivariable prediction model | Lauth 2015^42^ | No validated multivariable prediction model |
| Berger 2020^43^ | No validated multivariable prediction model | Lee 2023^44^ | No validated multivariable prediction model |
| Bergman 2011^45^ | No validated multivariable prediction model | Li 2017^46^ | No validated multivariable prediction model |
| Berloffa 2022^47^ | Other reason including design | Liang 2012^48^ | No validated multivariable prediction model |
| Bianchi 2023^49^ | No validated multivariable prediction model | Liu 2022^50^ | No validated multivariable prediction model |
| Brikell 2023^51^ | No validated multivariable prediction model | Liu 2007^52^ | Other reason including design |
| Brown 2012^53^ | No validated multivariable prediction model | Liu 2022^54^ | No validated multivariable prediction model |
| Cabana-Domínguez 2023^55^ | Other reason including design | Liu 2022^56^ | No validated multivariable prediction model |
| Cahn 1996^57^ | No validated multivariable prediction model | Liu 2021^58^ | No validated multivariable prediction model |
| Cai 2015^59^ | No validated multivariable prediction model | Liu 2021^60^ | No validated multivariable prediction model |
| Capusan 2016^61^ | No validated multivariable prediction model | Liu 2021^62^ | No validated multivariable prediction model |
| Catherine Joy 2022^63^ | No validated multivariable prediction model | Liu 2022^64^ | No validated multivariable prediction model |
| Cervantes-Henríquez 2022^65^ | No validated multivariable prediction model | Loftness 2023^66^ | Population |
| Cervantes-Henríquez 2022^67^ | Population | Loney 1978^68^ | No validated multivariable prediction model |
| Chandana 2018^69^ | No validated multivariable prediction model | Lopez-Villalobos 2014^70^ | No validated multivariable prediction model |
| Chang 2021^71^ | No validated multivariable prediction model | Lopez-Villalobos 2010^72^ | No validated multivariable prediction model |
| Chang 2022^73^ | No validated multivariable prediction model | Lopez-Villalobos 2010^74^ | No validated multivariable prediction model |
| Chang-Hyun 2021^75^ | Other reason including design | Lopez Villalobos 2011^76^ | No validated multivariable prediction model |
| Charach 2010^77^ | No validated multivariable prediction model | López-Villalobos 2010^78^ | No validated multivariable prediction model |
| Chauhan 2020^79^ | No validated multivariable prediction model | Luo 2020^80^ | No validated multivariable prediction model |
| Chauhan 2020^81^ | No validated multivariable prediction model | Mafi, 2022^82^ | Population |
| Chen 2019^83^ | No validated multivariable prediction model | Magee 2005^84^ | No validated multivariable prediction model |
| Chen 2019^85^ | No validated multivariable prediction model | Malegiannaki 2019^86^ | No validated multivariable prediction model |
| Chen 2020^87^ | No validated multivariable prediction model | Mancera Valetts 2015^88^ | No validated multivariable prediction model |
| Chen 2020^89^ | No validated multivariable prediction model | Moura 2017^90^ | No validated multivariable prediction model |
| Cheng 2012^91^ | No validated multivariable prediction model | Mueller 2010^92^ | No validated multivariable prediction model |
| Cho 2001^93^ | No validated multivariable prediction model | Munkvold 2014^94^ | No validated multivariable prediction model |
| Cicek 2018^95^ | No validated multivariable prediction model | Murugesan 2020^96^ | No validated multivariable prediction model |
| Cicek 2019^97^ | No validated multivariable prediction model | Nagai 2022^98^ | No validated multivariable prediction model |
| Cicek 2019^99^ | No validated multivariable prediction model | Østergaard 2016^100^ | No validated multivariable prediction model |
| Ciodaro 2020^101^ | No validated multivariable prediction model | Overgaard 2019^102^ | No validated multivariable prediction model |
| Colby 2012^103^ | No validated multivariable prediction model | Overgaard 2019^104^ | No validated multivariable prediction model |
| Cordova 2020^105^ | No validated multivariable prediction model | Oztoprak 2017^106^ | Other reason including design |
| Curtin 2022^107^ | No validated multivariable prediction model | Parashar 2021^108^ | No validated multivariable prediction model |
| Das 2021^109^ | No validated multivariable prediction model | Park 2023^110^ | No validated multivariable prediction model |
| Davis 2008^111^ | Other reason including design | Pastrana-Cortes 2021^112^ | No validated multivariable prediction model |
| De Dea 2019^113^ | Other reason including design | Paucke 2018^114^ | No validated multivariable prediction model |
| De Silva 2021^115^ | No validated multivariable prediction model | Peng 2013^116^ | Population |
| De Silva 2019^117^ | No validated multivariable prediction model | Pettersson 2018^118^ | No validated multivariable prediction model |
| Dimitrov 2023^119^ | No validated multivariable prediction model | Power 1998^120^ | No validated multivariable prediction model |
| Dincer 2012^121^ | No validated multivariable prediction model | Power 1998^122^ | No validated multivariable prediction model |
| Dou 2020^123^ | No validated multivariable prediction model | Predescu 2013^124^ | No validated multivariable prediction model |
| Du 2016^125^ | No validated multivariable prediction model | Qureshi 2016^126^ | Population |
| Dubreuil-Vall 2020^127^ | No validated multivariable prediction model | Qureshi 2017^128^ | Population |
| Duda 2017^129^ | No validated multivariable prediction model | Qureshi 2017^130^ | Population |
| Duda 2016^131^ | No validated multivariable prediction model | Rezaei 2022^132^ | Population |
| Efe 2022^133^ | Population | Riaz 2018^134^ | Population |
| Eloyan 2012^135^ | No validated multivariable prediction model | Richarte 2017^136^ | No validated multivariable prediction model |
| Eslami 2020^137^ | No validated multivariable prediction model | Rimvall 2014^138^ | No validated multivariable prediction model |
| Evans 2020^139^ | Population | Rishel 2005^140^ | No validated multivariable prediction model |
| Faraone 2021^141^ | No validated multivariable prediction model | Sabuncu 2015^142^ | Population |
| Fu 2013^143^ | No validated multivariable prediction model | Sachnev 2019^144^ | No validated multivariable prediction model |
| Fuermaier 2017^145^ | No validated multivariable prediction model | Sadatnezhad 2010^146^ | No validated multivariable prediction model |
| Fuermaier 2016^147^ | No validated multivariable prediction model | Shin 2023^148^ | No validated multivariable prediction model |
| Gabriel 2017^149^ | No validated multivariable prediction model | Shao 2018^150^ | Population |
| Gao 2009^151^ | No validated multivariable prediction model | Shao 2020^152^ | Population |
| Gao 2020^153^ | No validated multivariable prediction model | Shao 2019^154^ | Population |
| Gehricke 2017^155^ | No validated multivariable prediction model | Sharma 2014^156^ | Other reason including design |
| Gémes 2023^157^ | No validated multivariable prediction model | Shen 2021^158^ | No validated multivariable prediction model |
| Ghaderyan 2022^159^ | No validated multivariable prediction model | Shepler 2002^160^ | No validated multivariable prediction model |
| Ghasemi 2022^161^ | No validated multivariable prediction model | Sheriff 2022^162^ | No validated multivariable prediction model |
| Goh 2021^163^ | No validated multivariable prediction model | Solmaz 2012^164^ | Population |
| Gokten 2019^165^ | No validated multivariable prediction model | Son 2021^166^ | No validated multivariable prediction model |
| Grunblatt 2012^167^ | No validated multivariable prediction model | Sudre 2021^168^ | Population |
| Gu 2022^169^ | No validated multivariable prediction model | Tan 2017^170^ | Population |
| Guney 2021^171^ | No validated multivariable prediction model | Ter-Minassian 2022^172^ | No validated multivariable prediction model |
| Guntuku 2019^173^ | No validated multivariable prediction model | Thorell 2017^174^ | No validated multivariable prediction model |
| Guo 2022^175^ | No validated multivariable prediction model | Tran 2017^176^ | No validated multivariable prediction model |
| Gupta 2021^177^ | No validated multivariable prediction model | Trognon 2022^178^ | No validated multivariable prediction model |
| Gurevitz 2014^179^ | No validated multivariable prediction model | Uchida 2022^180^ | No validated multivariable prediction model |
| Hadas 2021^181^ | No validated multivariable prediction model | Uluyagmur-Ozturk 2016^182^ | No validated multivariable prediction model |
| Handen 1994^183^ | No validated multivariable prediction model | Ustun 2017^184^ | No validated multivariable prediction model |
| Hart 2014^185^ | No validated multivariable prediction model | Van der Meer 2017^186^ | No validated multivariable prediction model |
| Hazell 1999^187^ | No validated multivariable prediction model | Wang 2019^188^ | Population |
| Heller 2013^189^ | No validated multivariable prediction model | Wang 2020^190^ | Population |
| Hill 2009^191^ | Population | Wang 2017^192^ | Population |
| Hirvikoski 2021^193^ | Population | Wang 2022^194^ | No validated multivariable prediction model |
| Hu 2023^195^ | No validated multivariable prediction model | Wang 2023^196^ | Other reason including design |
| Huang 2022^197^ | Other reason including design | Wang 2023^198^ | Population |
| Iannaccone 2015^199^ | No validated multivariable prediction model | Wiguna 2022^200^ | No validated multivariable prediction model |
| Igual 2012^201^ | No validated multivariable prediction model | Yao 2018^202^ | Population |
| Ishizuya 2021^203^ | No validated multivariable prediction model | Zhang 2023^204^ | Population |
| Jafari 2022^205^ | No validated multivariable prediction model | Zhu 2023^206^ | No validated multivariable prediction model |
| Jansen 2021^207^ | Population |  |  |

**Table S6: Characteristics of diagnostic risk estimation models**

| **Author** | **Design** | **Setting** | **Age: mean±SD (range)** | **% females** | **Sample size** | **Outcome** | **Validation** | **Type of predictors** | **Discrimination: result (measure)**^a^ |
| --- | --- | --- | --- | --- | --- | --- | --- | --- | --- |
| Abibullaev 2012^208^ | Case-control | General population | (7-12) | N.a. | 10 | ADHD classification | Internal | EEG, cognitive | 0.9 (AUC) |
| Acosta-López 2021^209^ | Cohort | General population | 28.2±15.4 (8-60) | 44.8 | 232 | ADHD classification | Internal | Sociodemographic, cognitive | 0.8 (AUC) |
| Ahire 2023^210^ | Case-control | General population | (7-12) | N.a. | 121 | ADHD classification | Internal | EEG | 0.96 (accuracy) |
| Altinkaynak 2012^211^ | Case-control | Outpatient | (7-12) | 34.8 | 46 | ADHD classification | Internal | Cognitive | 0.91 (accuracy) |
| Altun 2022^212^ | Case-control | General population | (8-12) | 33.3 | 120 | ADHD classification | Internal | Clinical | 0.5-0.92 (AUC) |
| Altun 2022^213^ | Case-control | Outpatients | (8-12) | 31.7 | 120 | ADHD classification | Internal | Sociodemographic, clinical, cognitive | 0.833 (AUC) |
| Biederman 2017^214^ | Case-control | Outpatient | 30.0 (18-55) | 43 | 56 | ADHD classification | Internal | EEG, cognitive | Go condition: 0.92; No-Go condition: 0.84 (AUC) |
| Bledsoe 2016^215^ | Case-Control | General population | N.a. | 22.8 | 35 | ADHD classification | Internal | Clinical | 0.97 (AUC) |
| Bohland 2012^216^ | Case-control | General population | 12 (7–21) | 47 | 755 | ADHD classification | Internal | Neuroimaging | 0.78 (AUC) |
| Brown 2012^217^ | Case-control | General population | 12.4±3.3^b^ | 38^b^ | 973 | ADHD classification | Internal | Clinical, sociodemographic, neuroimaging | 0.62 (accuracy) |
| Caye 2020^218^ | Cohort | General population | N.a. | 51.2 | 5113 | ADHD classification | External | Clinical, sociodemographic, neuroimaging | 0.81 (AUC)^c^ |
| Cervantes-Henríquez 2022^219^ | Cohort | General population | 26.6±15.4 (6-60) | 43 | 480 | ADHD classification | Internal | Sociodemographic, genetic | 0.7 (AUC) |
| Cervantes-Henríquez 2020^220^ |  |  |  |  | 356 | ADHD classification | Internal | Sociodemographic, cognitive | 0.74 (AUC) |
| Chaim-Avancini 2017^221^ | Case-control | Outpatient | 27 | 24 | 116 | ADHD classification | Internal | Neuroimaging | 0.53-0.74 (AUC) |
| Chen 2019^222^ | Case-control | General population | 11.4±2.5 | 21.5 | 592 | ADHD classification | Internal | EEG | 0.93-0.98 (AUC) |
| Chen 2021^223^ | Cohort | N.a. | 33.0±-9.9 | 35 | 69 | ADHD classification | Internal | Sociodemographic, clinical, cognitive. | 0.56-0.79 (AUC) |
| Chen 2019^224^ | Case-control | Outpatient | 10.4±0.7 | 12 | 101 | ADHD classification | Internal | EEG | 0.95-0.98 (accuracy) |
| Cheng 2012^225^ | Case-control | General population | 12.1±2.0 | 28 | 239 | ADHD classification | Internal | Neuroimaging | 63.3; 85.1 (SENS; SPEC)^d^ |
| Chen 2022^226^ | Case-control | Outpatients | 12.0±3.3 | 35 | 872 | ADHD classification | Internal | Neuroimaging | 0.75 (AUC) |
| Chen 2023^227^ | Cohort | Outpatients | (17-72) | 35.7 | 501 | ADHD classification | Internal | Clinical | 0.80 (accuracy) |
| Christiansen 2020^228^ | Case-control | Outpatient | 32.4±9.9 | 39 | 977 | ADHD classification | Internal | Clinical, sociodemographic | 0.8 (AUC) |
| Chu2023^229^ | Case-control | Outpatients | 9.3 | N.a. | 95 | ADHD classification | Internal | Clinical | 0.848 (AUC) |
| Chugh2023^230^ | Case-control | General population | (7-26) | N.a. | 130 | ADHD classification | Internal, external | EEG | 0.988 (accuracy) |
| Crippa 2017^231^ | Case-Control | General population | 11.5 | 0 | 44 | ADHD classification | Internal | Cognitive, physical health, | 0.8 (AUC) |
| Dai 2012^232^ | Case-control | General population | 11.8±2.8 | 39 | 971 | ADHD classification | Internal | Neuroimaging | 0.59-0.71 (AUC) |
| DeLacy2023^233^ | Cohort | General population | (5.1-21.5) | 34.9 | 1120 | ADHD classification | Internal | Neuromaging | 0.95 (AUC) |
| Deserno 2022^234^ | Case-control | General population | 9.4±1.7 | 19 | 434 | ADHD classification | Internal | Clinical | 67; 84 (SENS; SPEC)^d^ |
| Dubreuil-Vall 2020^235^ | Case-Control | Outpatient | 43.8±14.8 | 50 | 40 | ADHD classification | Internal | EEG | 0.96 (AUC) |
| Duda 2016^236^ | Cohort | Outpatient | 11.2 | 37.3 | 2925 | Discrimination ADHD vs. ASD | Internal | Clinical | 0.96 (AUC) |
| Duda 2017^237^ | Case-Control | General population | 10.4 | 34.5 | 422 | DiscriminationADHD vs. ASD | Internal | Clinical | 0.89 (AUC) |
| Ehrig 2023^238^ | Case-control | Outpatients | 8.4±2.9 | 18.2 | 445 | Discrimination ADHD vs FASD | Internal | Sociodemographic, clinical, physical, cognitive | 0.92 (AUC) |
| Eloyan 2012^239^ | Case-control | General population | 12.4±3.3 | 38 | 970 | ADHD classification | Internal | Sociodemographic, neuroimaging^b^ | 0.61 (accuracy) |
| Emser 2018^240^ | Case-Control | Outpatient | 8.9±1.4 (children); 35.1±11.7 (adults) | 30 (children); 34.2 (adults) | 136 | ADHD classification | Internal | Sociodemographic, clinical, cognitive. | 83 Children, 90 adults (SENS), 90 (SPEC)^d^ |
| Esmailpoor 2015^241^ | Case-Control | NA | NA | 44 | 67 | ADHD classification | Internal | EEG | 100;97 (SENS; SPEC)^d^ |
| Finch 2015^242^ | Case-Control | Outpatient | 12.4±5.5 | 25.4 | 236 | ADHD classification | Internal | Cognitive | 67.8-96.6 (SENS); 77.1-95.8 (SPEC)^d^ |
| Garcia-Argibay 2022^243^ | Cohort | General population | (<18) | 33 | 238,696 | ADHD classification | Internal | Sociodemographic, clinical, cognitive, physical health | 0.75-0.8 (AUC) |
| Gaus 2022^244^ | Case-control | General population | 9.9±0.6 | 46.7 | 6916 | ADHD classification | Internal | Neuromaging | 0.567 (AUC) |
| Han 2020^245^ | Case control | Outpatient | (6-12) | 21.3 | 159 | ADHD classification | Internal | Clinical, cognitive | 0.82 (AUC) |
| Haque2023^246^ | Cohort | General population | (4-17) | N.a. | 1011 | Discrimination ADHD vs OCD vs SAD | Internal | Clinical | 0.91 (accuracy) |
| Hart 2013^247^ | Case-Control | Outpatient | 13.9±2 | 0 | 60 | ADHD classification | Internal | Neuroimaging | 0.81 (AUC) |
| Iannacone 2015^248^ | Case-Control | Outpatient | 14.5±1.5 | 39 | 36 | ADHD classification | Internal | Neuroimaging | 0.82 (AUC) |
| Itani 2019^249^ | Case-Control | Outpatient | 12.0±3.0 | 33.3-53.8 | 241 | ADHD classification | Internal | Sociodemographic, neuroimaging | 0.73 (accuracy) |
| Jahanshaloo 2022^250^ | Case-Control | General population | (4-15) | N.a. | 60 | ADHD classification | Internal | EEG | 0.98 (accuracy) |
| Johnston 2014^251^ | Case-Control | Outpatient | 12.5±2.3 | 0 | 68 | ADHD classification | Internal | Neuroimaging | 100; 85 (SENS; SPEC)^d^ |
| Kaur 2019^252^ | Case-Control | General population | 20.3±1.1 | 17 | 97 | ADHD classification | Internal | EEG | 0.93 (eyes-open) 0.90 (eyes-closed) 1.0 (CPT) (accuracy) |
| Kautzky 2020^253^ | Case-Control | Outpatient | 31.9±10.9 | 43.8 | 38 | ADHD classification | Internal | Genetic, neuroimaging | 82; 80 (SENS; SPEC)^d^ |
| Kiiski 2020^254^ | Case-Control | General population/Outpatient | 27.1±10.4 | 50 | 89 | ADHD classification | Internal | EEG | Eyes-Open: 0.57 Eyes-Closed: 0.6 (AUC) |
| Kim 2021^255^ | Case-Control | General population | 19.8±1.3 | 50.2 | 5726 | ADHD classification | Internal | Clinical | 0.78-0.81 (AUC) |
| Koh 2023^256^ | Case-control | Outpatients | (7-12) | 15.5 | 123 | Discrimination ADHD vs CD or both | Internal | ECG | 0.872 (accuracy) |
| Kurokami 2022^257^ | Case-control | Outpatients | (6-12) | 17.9 | 274 | ADHD classification | Internal | Clinical | Inattentive ADHD 0.884; combined ADHD 0.914 (AUC) |
| Lan 2021^258^ | Case-Control | Outpatient | 12.7±1.7 | 0 | 84 | ADHD classification | Internal | Neuroimaging | 0.72 (accuracy) |
| Lee 2008^259^ | Case-Control | Outpatient | 9.0±2.6 | 12.8 | 1938 | ADHD classification | Internal | Cognitive | 0.95 (accuracy) |
| Liang 2012^260^ | Case-Control | Outpatient | 11.2±2.7 | 20.5 | 149 | ADHD classification | Internal | Neuroimaging | 80.7, 79.5 (SENS; SPEC)^d^ |
| Lindheim 2022^261^ | Cohort | Web-based research registry | 9.6±1.6 | 40 | 30 | ADHD classification | Internal | Physical health | 0.89 (AUC) |
| Loh2023^262^ | Clinical trial | Outpatients | (7-16) | N.a. | 123 | ADHD classification | Internal | ECG | 0.96 (accuracy) |
| Lohani2023^263^ | Cohort | Outpatients | 11.6±3.1 | 47.8 | 632 | ADHD classification | Internal | Sociodemographic, clinical, neuroimaging | 0.75 (accuracy) |
| Luo 2020^264^ | Cohort | Outpatient | 24.4±2.1 | 14.9 | 72 | ADHD classification | Internal | Neuroimaging | 0.89 (AUC) |
| Maniruzzaman 2022^265^ | Case-control | General population | 9.6±1.8 | 13 | 121 | Current ADHD diagnosis | Internal | EEG | 0.96 (AUC) |
| Mao 2019^266^ | Case-control | General population | N.a. | N.a. | 788 | ADHD classification | Internal | Neuroimaging | 0.8 (AUC) |
| McNorman 2020^267^ | Case-control | General population | 24.0±1.3 | 20 | 80 | Current ADHD diagnosis | Internal | Neuroimaging | 0.91 (accuracy) |
| Mikolas 2022^268^ | Case-control | Secondary care outpatients | 10.0±2.4 | 13.7 | 299 | Classification of ADHD | Internal | Sociodemographic, clinical, cognitive | 0.66 (AUC) |
| Mock 2018^269^ | Cross-sectional | General population | (9-12) | 59.2 | 129 | Classification of ADHD | Internal | Cognitive | 0.91 (accuracy) |
| Moghaddari 2020^270^ | Case-control | General population | (7-12) | 23 | 61 | Current ADHD diagnosis | Internal | EEG | 0.98 (accuracy) |
| Mooney2023^271^ | Cohort | General population | 10.4±2.7 | 40.3 | 2480 | ADHD classification | Internal | Clinical, cognitive | 0.975 (accuracy) |
| Mueller 2010^272^ | Case-control | General population | 34.1±9.1 | 46 | 148 | Classification of ADHD | Internal | Cognitive, EEG | 0.92 (accuracy) |
| Mueller 2011^273^ |  |  | 36.0±8.4 | 49 | 150 | Classification of ADHD | External |  | 0.91 (accuracy) |
| Muller 2020^274^ | Case-control | General population | 32.1±12.0 | 58 | 328 | Current ADHD diagnosis | Internal | EEG | 0.84 (AUC) |
| Muthuraman 2019^275^ | Case-control | General population | 13.1±1.8 | 0 | 22 | Current ADHD diagnosis | Internal | EEG, EMG | 0.98 (accuracy) |
| Mwamba 2019^276^ | Case-control | General population | (5-16) | 53.3 | 30 | Current ADHD diagnosis | Internal | Cognitive, physical health | 0.83 (accuracy) |
| Oztekin 2021^277^ | Case-control | General population | 5.5 | N.a. | 162 | Current ADHD diagnosis | Internal | Clinical, cognitive | 0.94 (accuracy) |
| Oztekin 2022^278^ | Case-control | Outpatients | 5.5 (4-7) | N.a. | 180 | ADHD classification | Internal | Clinical, neuroimaging, cognitive | 0.922 (accuracy) |
| Oztoprak 2017^279^ | Case-control | General population | (6-12) | 0 | 108 | Current ADHD diagnosis | Internal | EEG | 0.98 (accuracy) |
| Pereda 2018^280^ | Case-control | Out/in patient | 8±0.3 | 0 | 33 | Current ADHD diagnosis | Internal | EEG | 0.94 (accuracy) |
| Saha 2022^281^ | Case-control | Na. | (0-22) | 31.3 | 220 | ADHD classification | Internal | Neuroimaging | 0.771 (AUC) females; 0.675 (AUC) males |
| Sen 2018^282^ | Case-control | General population | (0.1-29.5) | 37.5 | 1840 | Current ADHD diagnosis | Internal | Neuroimaging | 0.69 (accuracy) |
| Shi 2020^283^ | Cohort | general population | 16 (14–17) | 58 | 1593 | Classification of ADHD*** | Internal | Clinical | 0.95-0.98 (accuracy) |
| Silverstein 2016^284^ | Cohort within two RCTs | Primary care clinic | 8.7±2.1 | 31 | 156 | Current ADHD diagnosis | Internal | Clinical | 0.84 (accuracy) |
| Slobodin 2020^285^ | Case-control | Outpatient | 8.7±1.8 | 41 | 458 | Current ADHD diagnosis | Internal | Cognitive | 0.87 (accuracy) |
| Sun 2020^286^ | Case-control | General population | 11.8±2.9 | 0 | 68 | Current ADHD diagnosis | Internal | Neuroimaging | 0.85 (accuracy) |
| Tachmazidis 2021^287^ | Case-control | Outpatient | 33.0±9.9 | 65.2 | 69 | Current ADHD diagnosis | Internal | Clinical | 0.95 (accuracy) |
| Tenev 2014^288^ | Case-control | Outpatient | 33.4±8.4 | 49.2 | 117 | Classification of ADHD | Internal | EEG | 0.71-0.82 (accuracy)^e^ |
| Wang 2018^289^ | Case-control | Outpatient | 9.1 | 16.2 | 152 | Classification of ADHD | External | Genetic | 0.94 (AUC) |
| Yang2023^290^ | Case-control | Outpatients | 10.6±1.8 (8-15) | 8 | 72 | ADHD classification | Internal | Neuroimaging | 0.82 (accuracy) |
| Yasumura 2020^291^ | Case-control | Outpatient, general population | 10.6±2.2 | 16 | 315 | Classification of ADHD | Internal | Cognitive, sociodemographic, neuroimaging | 0.90 (AUC) |
| Yeh 2020^292^ | Case-control | General population | 8.6±1.5 | 38 | 68 | Classification of ADHD | Internal | Cognitive, clinical | 0.83 (accuracy) |
| Yoo 2019^293^ | Case-control | Outpatient | Training data set: 10.1±2.2; Validation: 9.4±2.4 | Training: 21.3; Validation:33.3 | 130 | Classification of ADHD | Internal, external | Neuroimaging, genetic | 0.89 (AUC), 0.65 (AUC) |
| Zhang-James 2021^294^ | Case-control | General population | (4-63) | 58.7 | 4042 | Classification of ADHD | Internal | Sociodemographic, neuroimaging | 0.63 (AUC) |
| Zhu 2022^295^ | Case-control | Outpatient (cases), general population (controls) | 8.2±1.8 | 17.5 | 682 | Classification of ADHD | External | Genetic | 0.94 (AUC) |

^a^Accuracy typically represented as % in the included studies; ^b^Data from Brown and Eloyan 2012 obtained from either PDF as similar sample; ^c^Bias corrected; ^d^Data about sensitivity and specificity (in %) reported as accuracy/AUC not reported; ^e^Eyes open: 70.9%, eyes closed: 72.6%, VCPT: 69.2%, ECPT: 71.7%, voter: 82.3%; ^f^And learning difficulties.

ADHD: Attention Deficit and Hyperactivity Disorder; AUC: Area Under Curve; CPT: Continuous Performance Test; EEG: Electroencephalogram; EMG: Electromynogram; N.a: not available; SENS: sensitivity; SPEC: specificity.

**Table S7: Characteristics of prognostic risk estimation models**

| **Author** | **Design** | **Setting** | **Age: mean±SD (range)** | **% females** | **Sample size** | **Outcome** | **Validation** | **Type of predictors** | **Discrimination: result (measure)**^a^ |
| --- | --- | --- | --- | --- | --- | --- | --- | --- | --- |
| Franz 2022^296^ | Case-control | Inpatient | Newborns | 47 | 96 | Development of ADHD. | Internal | Physical health, clinical | 0.87 (AUC) |
| Lavigne 2023^297^ | Cohort | General population | 4.4 | 50.1 | 775 | Stability of diagnosis | Internal | Sociodemographic, clinical | 65.8% better than chance prediction |
| Suresh 2021^298^ | Cohort | ADHD patients | 16.3 | 44 | 77 | Symptom change. | Internal | Neuroimaging, genetic | 0.46-0.64 (R) |
| Zhang 2020^299^ | Cohort | Inpatients | 30.4±7.3 | 28 | 2639 | Psychiatric symptoms. | Internal, external | Clinical, genetic | 0.75 (AUC), 0.75 (AUC) |
| Zhang-James 2020^300^ | Cohort | Outpatient and inpatient | N.a. | N.a. | 19,787 | Development of SUD. | Internal | Sociodemographic, clinical | 0.73 (AUC) |

^a^Accuracy typically represented as % in the included studies;

ADHD: Attention Deficit and Hyperactivity Disorder; AUC: Area Under Curve; SUD: substance use disorders.

Evidence for similar structural brain anomalies in youth and adult attention-deficit/hyperactivity disorder:

https://link.springer.com/article/10.1007/s13755-020-00123-7#Tab3

**Table S8: Characteristics of predictive risk estimation models**

| **Author** | **Design** | **Setting** | **Age: mean±SD (range)** | **% females** | **Sample size** | **Outcome** | **Validation** | **Type of predictors** | **Discrimination: result (measure)**^a^ |
| --- | --- | --- | --- | --- | --- | --- | --- | --- | --- |
| Chang 2021^301^ | Cohort | Outpatient | 17.5±9.8 (6-42) | N.a. | 79 | Good/Poor responders (CGI-I) | Internal | Neuroimaging | 0.88 (AUC) |
| Faraone 2021^302^ | RCT | Outpatient | 11.0±3.2 (6-17) | NA | 774 | Treatment response | Internal | Sociodemographic, clinical, cognitive, physical health | 0.84 (AUC) |
| Kim 2015^303^ | Open-label trial | Outpatient | 9.6±2.5 | 20.5 | 78 | Internal | Internal | Genetic, cognitive, physical health | 0.84 (AUC) |
| Morrow 2020^304^ | Cohort | General population | 12.4±3.4 | 31.5 | 6630 | Receipt of psychosocial treatment for ADHD. | Internal | Clinical, sociodemographic, service use, physical health | 0.72 (AUC) |
| Setyawan 2015^305^ | Cohort within RCT | Ourtpatient | 10.8±2.8 | 20.8 | 211 | Treatment failure | Internal | Sociodemographic, clinical | 0.86 (AUC) |
| Wang 2022^306^ | Case-control | Outpatients | 8.9±2.2 | 23.4 | 228 | Treatment response | Internal | Genetic | 0.996 (AUC) |
| Wong 2017^307^ | Cohort | Outpatient | (6–17) | N.a. | 157 | Remission classification | Internal | Sociodemographic, clinical, physical health | 0.83 (AUC) |

^a^Accuracy typically represented as % in the included studies.

ADHD: Attention Deficit and Hyperactivity Disorder; AUC: Area Under Curve; RCT: randomized clinical trial.

**Table S9: PROBAST Quality assessment results**^a^

|  | **Participants**  **Risk of bias** | **Predictors**  **Risk of bias** | **Outcomes**  **Risk of bias** | **Analysis**  **Risk of bias** | **Overall**  **Risk of bias** |  |  |
| --- | --- | --- | --- | --- | --- | --- | --- |
| Abibullaev 2012^208^ | High | High | Low | High | High |  |  |
| Acosta-López 2021^209^ | Low | Low | Low | Low | Low |  |  |
| Ahire 2023^210^ | | Low | Unclear | Low | Low | Unclear |  |
| Altinkaynak 2012^211^ | Low | High | Low | Low | High |  |  |
| Altun 2022^212^ | Low | High | Low | Low | High |  |  |
| Altun 2022^213^ | Low | Unclear | Low | Unclear | Unclear |  |  |
| Biederman 2017^214^ | Low | Low | Low | Low | Low |  |  |
| Bledsoe 2016^215^ | Low | High | Low | High | High |  |  |
| Bohland 2012^216^ | Low | Low | Unclear | Unclear | Unclear |  |  |
| Brown 2012^217^ | Low | Low | Unclear | Unclear | Unclear |  |  |
| Caye 2020^218^ | Low | Low | Low | Low | Low |  |  |
| Cervantes-Henríquez 2022^219^ | Low | Low | Low | Low | Low |  |  |
| Cervantes-Henríquez 2020^220^ | Low | Low | Low | Low | Low |  |  |
| Chaim-Avancini 2017^221^ | Low | Low | Low | High | High |  |  |
| Chang 2021^301^ | Low | Low | Low | Low | Low |  |  |
| Chen 2019^224^ | Low | Low | High | High | High |  |  |
| Chen 2019.2^222^ | Low | Low | Unclear | Unclear | Unclear |  |  |
| Chen 2021^223^ | High | Low | High | Low | High |  |  |
| Chen 2022^226^ | | | Low | Unclear | Low | High | High |
| Chen 2023^227^ | | | Low | Unclear | Unclear | High | Unclear |
| Cheng 2012^225^ | Unclear | Low | Low | High | High |  |  |
| Chu2023^229^ | Low | Unclear | Unclear | Unclear | High |  |  |
| Chugh2023^230^ | Low | Unclear | Low | Low | Unclear |  |  |
| Christiansen 2020 ^228^ | Low | Low | High | High | High |  |  |
| Crippa 2017^231^ | High | Unclear | Low | High | High |  |  |
| Dai 2012^232^ | High | Low | Low | High | High |  |  |
| DeLacy2023^233^ | Low | Unclear | Low | Low | Unclear |  |  |
| Deserno 2022^234^ | Low | Low | High | High | High |  |  |
| Dubreuil-Vall 2020^235^ | Low | Low | Low | High | High |  |  |
| Duda 2016^236^ | Low | High | High | Low | High |  |  |
| Duda 2017^237^ | Low | Low | High | High | High |  |  |
| Ehrig 2023^238^ | Low | Unclear | Low | Unclear | Unclear |  |  |
| Eloyan 2012^239^ | Low | Low | Unclear | High | High |  |  |
| Emser 2018^240^ | High | High | High | High | High |  |  |
| Esmailpoor 2015^241^ | High | High | Low | High | High |  |  |
| Faraone 2021^302^ | Low | Low | Low | Low | Low |  |  |
| Finch 2015^242^ | Low | Low | Low | High | High |  |  |
| Franz 2022^296^ | Low | Low | Low | High | High |  |  |
| Garcia-Argibay 2022 ^243^ | Low | Low | Low | Unclear | Unclear |  |  |
| Gaus 2022^244^ | Low | Unclear | Low | High | High |  |  |
| Han 2020^245^ | High | Unclear | Unclear | High | High |  |  |
| Haque2023^246^ | Low | Unclear | Low | Low | Unclear |  |  |
| Hart 2014^247^ | Low | Low | Low | High | High |  |  |
| Iannacone 2015^248^ | Low | Low | Low | High | High |  |  |
| Itani 2019^249^ | Low | Low | Low | High | High |  |  |
| Jahanshaloo 2017^250^ | Unclear | Low | High | High | High |  |  |
| Johnston 2014^251^ | Low | Low | Low | High | High |  |  |
| Kaur 2020^252^ | Low | Low | Low | High | High |  |  |
| Kautzky 2020^253^ | Low | Low | Low | High | High |  |  |
| Kiiski 2020^254^ | Low | Low | High | High | High |  |  |
| Kim 2015^303^ | Low | Low | Low | High | High |  |  |
| Kim 2021^255^ | Unclear | Low | High | High | High |  |  |
| Koh 2023^256^ | Low | Unclear | Low | Low | Unclear |  |  |
| Kurokami 2022^257^ | Low | Unclear | Low | Unclear | Unclear |  |  |
| Lan 2021^258^ | Low | Low | Low | High | High |  |  |
| Lavigne 2023^297^ | Low | Unclear | Low | High | High |  |  |
| Lee 2008^259^ | High | Low | High | High | High |  |  |
| Liang 2012^260^ | Low | Low | Low | High | High |  |  |
| Lindheim 2022^261^ | Low | Low | High | High | High |  |  |
| Loh2023^262^ | Low | Unclear | Low | Unclear | Unclear |  |  |
| Lohani2023^263^ | Low | Unclear | Low | Unclear | Unclear |  |  |
| Luo 2020^264^ | Low | High | High | High | High |  |  |
| Maniruzzaman 2022^265^ | Low | Unclear | Unclear | High | High |  |  |
| Mao 2019^266^ | Unclear | Low | Unclear | High | High |  |  |
| McNorman 2020^267^ | Low | Unclear | High | High | High |  |  |
| Mikolas 2022^268^ | Low | High | High | High | High |  |  |
| Mock 2018^269^ | Low | Unclear | Unclear | High | High |  |  |
| Moghaddari 2020^270^ | Low | Low | Unclear | Unclear | Unclear |  |  |
| Mooney2023^271^ | Low | Unclear | Low | Low | Unclear |  |  |
| Morrow 2020^304^ | Low | Unclear | Unclear | High | High |  |  |
| Mueller 2010^272^ | Low | Low | Unclear | Unclear | Unclear |  |  |
| Mueller 2011^273^ | Low | Unclear | Unclear | High | High |  |  |
| Muller 2020^274^ | Low | Low | Unclear | Unclear | Unclear |  |  |
| Muthuraman 2019^275^ | Low | Low | Unclear | Unclear | Unclear |  |  |
| Mwamba 2019^276^ | Low | Unclear | Unclear | High | High |  |  |
| Oztekin 2021^277^ | High | Low | Unclear | Unclear | Unclear |  |  |
| Oztekin 2022^278^ | Low | Unclear | Low | High | High |  |  |
| Oztoprak 2017^279^ | Low | Low | Unclear | High | High |  |  |
| Pereda 2018^280^ | Low | Low | Unclear | High | High |  |  |
| Saha 2022^281^ | Low | Unclear | High | High | High |  |  |
| Sen 2018^282^ | Low | Low | Low | High | High |  |  |
| Setyawan 2015^305^ | Low | Low | Low | High | High |  |  |
| Silverstein 2016 ^284^ | Low | Low | High | High | High |  |  |
| Slobodin 2020^285^ | Low | Unclear | Low | High | High |  |  |
| Shi 2020^283^ | Low | High | High | Unclear | High |  |  |
| Sun 2020^286^ | Low | Low | Unclear | Unclear | Unclear |  |  |
| Suresh 2021^298^ | Low | Low | Low | Unclear | Unclear |  |  |
| Tachmazidis 2021^287^ | Low | High | Unclear | High | High |  |  |
| Tenev 2014^288^ | Low | Low | High | Unclear | High |  |  |
| Wang 2018^289^ | Low | Low | Unclear | High | High |  |  |
| Wang 2022^306^ | Low | Unclear | Low | High | High |  |  |
| Wong 2017^307^ | Low | Low | Low | Unclear | Unclear |  |  |
| Yang2023^290^ | Low | Unclear | Low | High | High |  |  |
| Yasumura 2020^291^ | Low | Low | Unclear | High | High |  |  |
| Yeh 2020^292^ | Low | Low | Low | High | High |  |  |
| Yoo 2019^293^ | Low | Low | Low | High | High |  |  |
| Zhang 2020^299^ | Low | Low | Low | High | High |  |  |
| Zhang-James 2020^300^ | Low | Low | Low | High | High |  |  |
| Zhang-James 2021^294^ | Low | Low | Low | Low | Low |  |  |
| Zhu 2022^295^ | Unclear | Low | Low | Unclear | Unclear |  |  |

^a^To be considered at low risk of bias all questions should be answered as appropriate^308, 309^

**Table S10. Statistical evaluation of validation features**^a^**.**

|  | **Double dipping (evaluating performance in the development dataset) detected** | **Reporting of apparent predictive performance instead of cross-validated predictive performance detected.** | **Reporting of size and significance of regression coefficients instead of cross-validated performance measure detected.** | **Re-estimation regression coefficients in the test set detected.** | **Overall suboptimal validation detected.** |
| --- | --- | --- | --- | --- | --- |
| Abibullaev 2012^208^ | Yes | No | No | No | Yes |
| Acosta-López 2021^209^ | No | No | No | No | No |
| Ahire 2023^210^ | No | No | No | No | No |
| Altinkaynak 2012^211^ | Yes | No | No | No | Yes |
| Altun 2022^212^ | No | No | No | No | No |
| Altun 2022^213^ | No | No | No | No | No |
| Biederman 2017^214^ | No | No | No | No | No |
| Bledsoe 2016^215^ | No | No | No | No | No |
| Bohland 2012^216^ | No | No | No | No | No |
| Brown 2012^217^ | No | No | No | No | No |
| Caye 2020^218^ | No | No | No | No | No |
| Cervantes-Henríquez 2022^219^ | No | No | No | No | No |
| Cervantes-Henríquez 2020^220^ | No | No | No | No | No |
| Chaim-Avancini 2017^221^ | No | No | No | No | No |
| Chang 2021^301^ | No | No | No | No | No |
| Chen 2019^224^ | No | No | No | No | No |
| Chen 2019.2^222^ | No | No | No | No | No |
| Chen 202^223^ | No | No | No | No | No |
| Cheng 2012^225^ | No | No | No | No | No |
| Chen 2022^226^ | No | No | No | No | No |
| Chen 2023^227^ | No | No | No | No | No |
| Christiansen 2020^228^ | No | No | No | No | No |
| Chu2023^229^ | No | No | No | No | No |
| Chugh2023^230^ | No | No | No | No | No |
| Crippa 2017^231^ | No | No | No | No | No |
| Dai 2012^232^ | No | No | No | No | No |
| DeLacy2023^233^ | No | No | No | No | No |
| Deserno 2022^234^ | No | No | No | No | No |
| Dubreuil-Vall 2020^235^ | No | No | No | No | No |
| Duda 2016^236^ | No | No | No | No | No |
| Duda 2017^237^ | No | No | No | No | No |
| Ehrig 2023^238^ | No | No | No | No | No |
| Eloyan 2012^239^ | No | No | No | No | No |
| Emser 2018^240^ | No | No | No | No | No |
| Esmailpoor 2015^241^ | Yes | No | No | No | Yes |
| Faraone 2021^302^ | No | No | No | No | No |
| Finch 2015^242^ | No | No | No | No | No |
| Franz 2022^296^ | No | No | No | No | No |
| Garcia-Argibay 2022^243^ | No | No | No | No | No |
| Gaus 2022^244^ | No | No | No | No | No |
| Han 2020^245^ | Yes | No | No | No | Yes |
| Haque2023^246^ | No | No | No | No | No |
| Hart 2014^247^ | No | No | No | No | No |
| Iannacone 2015^248^ | No | No | No | No | No |
| Itani 2019^249^ | No | No | No | No | No |
| Jahanshaloo 2017^250^ | No | No | No | No | No |
| Johnston 2014^251^ | No | No | No | No | No |
| Kaur 2020^252^ | Yes | No | No | No | Yes |
| Kautzky 2020^253^ | No | No | No | No | No |
| Kiiski 2020^254^ | No | No | No | No | No |
| Kim 2015^303^ | No | No | No | No | No |
| Kim 2021^255^ | No | No | No | No | No |
| Koh 2023^256^ | Yes | No | No | No | Yes |
| Kurokami 2022^257^ | No | No | No | No | No |
| Lan 2021^258^ | No | No | No | No | No |
| Lavigne 2023^297^ | No | No | No | No | No |
| Lee 2008^259^ | No | No | No | No | No |
| Liang 2012^260^ | No | No | No | No | No |
| Lindheim 2022^261^ | Yes | No | No | No | Yes |
| Loh2023^262^ | No | No | No | No | No |
| Lohani2023^263^ | Yes | No | No | No | Yes |
| Luo 2020^264^ | Yes | No | No | No | Yes |
| Maniruzzaman 2022^265^ | Yes | No | No | No | Yes |
| Mao 2019^266^ | No | No | No | No | No |
| McNorman 2020^267^ | No | No | No | No | No |
| Mikolas 2022^268^ | No | No | No | No | No |
| Mock 2018^269^ | No | No | No | No | No |
| Moghaddari 2020^270^ | No | No | No | No | No |
| Morrow 2020^304^ | No | No | No | No | No |
| Mooney2023^271^ | No | No | No | No | No |
| Mueller 2010^272^ | No | No | No | No | No |
| Mueller 2011^273^ | No | No | No | No | No |
| Muller 2020^274^ | No | No | No | No | No |
| Muthuraman 2019^275^ | No | No | No | No | No |
| Mwamba 2019^276^ | No | No | No | No | No |
| Oztekin 2021^277^ | No | No | No | No | No |
| Oztekin 2022^278^ | No | No | No | No | No |
| Oztoprak 2017^279^ | No | No | No | No | No |
| Pereda 2018^280^ | Yes | No | No | No | Yes |
| Saha 2022^281^ | No | No | No | No | No |
| Sen 2018^282^ | No | No | No | No | No |
| Setyawan 2015^305^ | No | No | No | No | No |
| Silverstein 2016^284^ | No | No | No | No | No |
| Slobodin 2020^285^ | No | No | No | No | No |
| Shi 2020^283^ | No | No | No | No | No |
| Sun 2020^286^ | No | No | No | No | No |
| Suresh 2021^298^ | No | No | No | No | No |
| Tachmazidis 2021^287^ | No | No | No | No | No |
| Tenev 2014^288^ | Yes | No | No | No | Yes |
| Wang 2022^306^ | Yes | No | No | No | Yes |
| Wang 2018^289^ | No | No | No | No | No |
| Wong 2017^307^ | No | No | No | No | No |
| Yang2023^290^ | No | No | No | No | No |
| Yasumura 2020^291^ | No | No | No | No | No |
| Yeh 2020^292^ | No | No | No | No | No |
| Yoo 2019^293^ | No | No | No | No | No |
| Zhang 2020^299^ | No | No | No | No | No |
| Zhang-James 2020^300^ | No | No | No | No | No |
| Zhang-James 2021^294^ | No | No | No | No | No |
| Zhu 2022^295^ | No | No | No | No | No |

^a^ To note, we could only evaluate the information reported by the individual studies/authors. we cannot discard suboptimal validation strategies carried out but not reported by individual authors.

**Supplement 1: Literature search**

The following search terms were applied:

(“risk calculator” OR “risk prediction” OR “prediction model” OR “predictive model” OR “prognostic model” OR “diagnostic model” OR “individualised risk” OR “random forest” OR “neural network” OR “classification tree” OR “regression tree” OR “estimation tree” OR “elastic net” OR “deep learning” OR “estimation algorithm” OR “machine learning” OR “vector machine” OR “boosting” OR “lasso” OR “ridge” OR “regression” OR “logistic regression” OR “cox regression”) AND (“ADHD” OR “Attention Deficit” OR “Attention-deficit” OR “AttentionDeficit/Hyperactivity disorder” OR “hyperkinetic disorder” OR “hyperkinetic syndrome”).

**Supplement 2: Variables included in the study**

The following variables were included from the “Checklist for critical Appraisal and data extraction for systematic Reviews of prediction Modelling Studies” (CHARMS)^310^: First author and year of publication; Study type (diagnostic, prognostic, predictive); Mean age; Sex; Time points; Statistical analysis; Model presentation; Source of data (cohort, case control, clinical trial or registry data); Setting; Participant eligibility and recruitment method; Participant description; Treatments received; Study dates; Definition and method for measurement of outcome; Type of outcome (single or multiple endpoints); Predictors part of outcome or not; Time of outcome occurrence; Number of predictors; Type of predictors (neuroimaging, cognitive, sociodemographic, electroencephalographic, physical health, genetic, magnetoencephalographic, service use actigraphic - actigraphic predictor category has been introduced post-protocol and was not included in our previous study-) Selection of predictors; Sample size; Number of events; Quantity of missing data; Handling of missing data; Model development; Modelling method; Methods for selection of predictors; Calibration measures; Discrimination measures and results; Classification measures (sensitivity, specificity, predictive values); Method used for testing model performance; Blinding of Predictors; Handling of Predictors; Satisfaction of Modelling assumptions satisfied or not; Shrinkage of predictor weighs or regression coefficients; Calibration validation; Type of validation (internal and external); Discrimination measures and results (derivation and external validation); Model adjustment (if applicable); Multivariable model (predictors included); Interpretation of presented models; Discussion of the results.

**Supplement 3: Quality assessment and risk of bias items (PROBAST v5/05/2019**^309, 311^**)**

PROBAST includes four steps: specify the systematic review question; classify the type of prediction model evaluation (e.g. internal or external validation; assess risk of bias and applicability of core domains (participants, predictors, outcome, and analysis) and overall judgement (low, high or unclear)^311^.

The review question is detailed in the introduction of this manuscript; the type of prediction model for each of the studies is detailed in the methods; risk of bias and applicability were assessed with the PROBAST questions (see below). We finally classified the models as “low risk/ high risk” for each of the domains. To be considered at low risk of bias all questions should be answered as appropriate (yes or probably yes)^308, 309^. An outcome is considered to be at high risk of bias when at least one of the questions is answered as not appropriate (no or probably no). The overall risk of bias is considered high risk when one or more domains is considered at high risk^308^.

Step 3 PROBAST Questions:

**Participants:**

- 1. Were appropriate data sources used, e.g. cohort, RCT or nested case-control study data?
  2. Were all inclusions and exclusions of participants appropriate?

**Predictors:**

- 1. Were predictors defined and assessed in a similar way for all participants?
  2. Were predictor assessments made without knowledge of outcome data?
  3. Are all predictors available at the time the model is intended to be used?

**Outcomes:**

- 1. Was the outcome determined appropriately?
  2. Was a pre-specified or standard outcome definition used?
  3. Were predictors excluded from the outcome definition?
  4. Was the outcome defined and determined in a similar way for all participants?
  5. Was the outcome determined without knowledge of predictor information?
  6. Was the time interval between predictor assessment and outcome determination appropriate?

**Analysis:**

- 1. Were there a reasonable number of participants with the outcome?
  2. 4.2 Were continuous and categorical predictors handled appropriately?
  3. Were all enrolled participants included in the analysis?
  4. Were participants with missing data handled appropriately?
  5. Was selection of predictors based on univariable analysis avoided?
  6. Were complexities in the data (e.g. censoring, competing risks, sampling of controls) accounted for appropriately?
  7. Were relevant model performance measures evaluated appropriately?
  8. Were model overfitting and optimism in model performance accounted for?

4.9 Do predictors and their assigned weights in the final model correspond to the results from multivariable analysis?

**REFERENCES**

1. Makris G, Pervanidou P, Chouliaras G, Stachtea X, Valavani E, Bastaki D *et al.* Diverse patterns of vulnerability to visual illusions in children with neurodevelopmental disorders. *Cogn Process* 2021; **22**(4)**:** 659-673.

2. Snell KIE, Levis B, Damen JAA, Dhiman P, Debray TPA, Hooft L *et al.* Transparent reporting of multivariable prediction models for individual prognosis or diagnosis: checklist for systematic reviews and meta-analyses (TRIPOD-SRMA). *BMJ* 2023; **381:** e073538.

3. Abello V, Mantilla WA, Idrobo H, Sossa CL, Salazar LA, Pena A *et al.* Real-World Evidence of Epidemiology and Clinical Outcomes in Multiple Myeloma, Findings from the Registry of Hemato-Oncologic Malignancies in Colombia, Observational Study. *Clin Lymphoma Myeloma Leuk* 2022; **22**(6)**:** e405-e413.

4. Johnston BA, Mwangi B, Matthews K, Coghill D, Konrad K, Steele JD. Brainstem abnormalities in attention deficit hyperactivity disorder support high accuracy individual diagnostic classification. *Hum Brain Mapp* 2014; **35**(10)**:** 5179-5189.

5. Aceves-Fernandez MA. Methodology proposal of ADHD classification of children based on cross recurrence plots. *Nonlinear Dynamics* 2021; **104**(2)**:** 1491-1505.

6. Joshi AA, Choi S, Li J, Akrami H, Leahy RM, Spie. *A Pairwise Approach for fMRI Group Studies using the BrainSync Transform*, vol. 115962021.

7. Agarwal C, Gupta S, Najjar M, Weaver TE, Zhou XJ, Schonfeld D *et al.* Deep Learning Analyses of Brain MRI to Identify Sustained Attention Deficit in Treated Obstructive Sleep Apnea: A Pilot Study. *Sleep Vigil* 2022; **6**(1)**:** 179-184.

8. Karam RG, Breda V, Picon FA, Rovaris DL, Victor MM, Salgado CA *et al.* Persistence and remission of ADHD during adulthood: a 7-year clinical follow-up study. *Psychol Med* 2015; **45**(10)**:** 2045-2056.

9. Ahmadi A, Kashefi M, Shahrokhi H, Nazari MA. Computer aided diagnosis system using deep convolutional neural networks for ADHD subtypes. *Biomedical Signal Processing and Control* 2021; **63**.

10. Kaur S, Singh S, Arun P, Kaur D, Jadavpur Univ Main C, Ieee *et al.* *EEG Based Decision Support System to Diagnose Adults with ADHD*, 2018, 87-91pp.

11. Ahmadlou M, Adeli H. Wavelet-synchronization methodology: a new approach for EEG-based diagnosis of ADHD. *Clin EEG Neurosci* 2010; **41**(1)**:** 1-10.

12. Kaur A, Kahlon KS. Accurate Identification of ADHD among Adults Using Real-Time Activity Data. *Brain Sci* 2022; **12**(7).

13. Amado-Caballero P, Casaseca-de-la-Higuera P, Alberola-Lopez S, Andres-de-Llano JM, Villalobos JAL, Garmendia-Leiza JR *et al.* Objective ADHD Diagnosis Using Convolutional Neural Networks Over Daily-Life Activity Records. *IEEE J Biomed Health Inform* 2020; **24**(9)**:** 2690-2700.

14. Kessler RC, Adler LA, Barkley R, Biederman J, Conners CK, Faraone SV *et al.* Patterns and predictors of attention-deficit/hyperactivity disorder persistence into adulthood: results from the national comorbidity survey replication. *Biol Psychiatry* 2005; **57**(11)**:** 1442-1451.

15. Alameda L, Christy A, Rodriguez V, Salazar de Pablo G, Thrush M, Shen Y *et al.* Association Between Specific Childhood Adversities and Symptom Dimensions in People With Psychosis: Systematic Review and Meta-Analysis. *Schizophr Bull* 2021; **47**(4)**:** 975-985.

16. Khoshnoud S, Shamsi M, Nazari MA, Ieee. *Non-linear EEG Analysis in Children with Attention-Deficit/Hyperactivity Disorder During the Rest Condition*, 2015, 87-92pp.

17. An H, Wei C-s, Wang O, Wang D-h, Xu L-w, Lu Q *et al.* An ensemble-based likelihood ratio approach for family-based genomic risk prediction. *Journal of Zhejiang University-Science B* 2018; **19**(12)**:** 935-947.

18. Kiiski H, Rueda-Delgado LM, Bennett M, Knight R, Rai L, Roddy D *et al.* Functional EEG connectivity is a neuromarker for adult attention deficit hyperactivity disorder symptoms. *Clin Neurophysiol* 2020; **131**(1)**:** 330-342.

19. Anderson A, Douglas PK, Kerr WT, Haynes VS, Yuille AL, Xie J *et al.* Non-negative matrix factorization of multimodal MRI, fMRI and phenotypic data reveals differential changes in default mode subnetworks in ADHD. *Neuroimage* 2014; **102 Pt 1:** 207-219.

20. Kiiski H, Bennett M, Rueda-Delgado LM, Farina FR, Knight R, Boyle R *et al.* EEG spectral power, but not theta/beta ratio, is a neuromarker for adult ADHD. *Eur J Neurosci* 2020; **51**(10)**:** 2095-2109.

21. Ardulov V, Martinez VR, Somandepalli K, Zheng S, Salzman E, Lord C *et al.* Robust diagnostic classification via Q-learning. *Sci Rep* 2021; **11**(1)**:** 11730.

22. Kim MG, Kim J, Kim SC, Jeong J. Twitter Analysis of the Nonmedical Use and Side Effects of Methylphenidate: Machine Learning Study. *J Med Internet Res* 2020; **22**(2)**:** e16466.

23. Areces D, Rodriguez C, Garcia T, Cueli M. Is an ADHD Observation-Scale Based on DSM Criteria Able to Predict Performance in a Virtual Reality Continuous Performance Test? *Applied Sciences-Basel* 2020; **10**(7).

24. Kooij JJS, Roesler M, Philipsen A, Waechter S, Dejonckheere J, van der Kolk A *et al.* Predictors and impact of non-adherence in adults with attention-deficit/hyperactivity disorder receiving OROS methylphenidate: results from a randomized, placebo-controlled trial. *BMC Psychiatry* 2013; **13**.

25. Aydin S, Cetin FH, Uytun MC, Babadagi Z, Gueven AS, Isik Y. Comparison of domain specific connectivity metrics for estimation brain network indices in boys with ADHD-C. *Biomedical Signal Processing and Control* 2022; **76**.

26. Kuang D, Guo X, An X, Zhao Y, He L, Soc ICI *et al.* *Discrimination of ADHD Based on fMRI Data with Deep Belief Network*, vol. 85902014, 225-232pp.

27. Balboni G, Incognito O, Belacchi C, Bonichini S, Cubelli R. Vineland-II adaptive behavior profile of children with attention-deficit/hyperactivity disorder or specific learning disorders. *Res Dev Disabil* 2017; **61:** 55-65.

28. Kushki A, Anagnostou E, Hammill C, Duez P, Brian J, Iaboni A *et al.* Examining overlap and homogeneity in ASD, ADHD, and OCD: a data-driven, diagnosis-agnostic approach. *Transl Psychiatry* 2019; **9**(1)**:** 318.

29. Basaran M, Tosun M, Kabay SC, Akdag G. Estimation Of Attention Deficit And Hyperactivity Disorder (ADHD) With Artificial Neural Networks Using EEG Signals. *European Journal of Neurology* 2020; **27:** 822-822.

30. Kurokami T, Kobayashi H, Nakajima M, Mikami M, Koeda T. Establishment of an objective index for the diagnosis of attention deficit/hyperactivity disorder by the continuous performance test "MOGRAZ". *Brain Dev* 2022.

31. Bashiri A, Shahmoradi L, Beigy H, Savareh BA, Nosratabadi M, Kalhori SRN *et al.* Quantitative EEG features selection in the classification of attention and response control in the children and adolescents with attention deficit hyperactivity disorder. *Future Science Oa* 2018; **4**(5).

32. Labarga SZ, Hoberg K, Hamadache S, Guenther T. Validation of the QbMini Test to diagnose Attention Deficit and Hyperactivity Disorder (ADHD) in 5-year-old children. *Zeitschrift Fur Neuropsychologie* 2019; **30**(3)**:** 149-156.

33. Guadalupe Bedolla-Ibarra M, del Carmen Cabrera-Hernandez M, Antonio Aceves-Fernandez M, Tovar-Arriaga S. Classification of attention levels using a Random Forest algorithm optimized with Particle Swarm Optimization. *Evolving Systems* 2022.

34. Lan Z, Sun Y, Zhao L, Xiao Y, Kuai C, Xue S-W. Aberrant Effective Connectivity of the Ventral Putamen in Boys With Attention-Deficit/Hyperactivity Disorder. *Psychiatry Investigation* 2021; **18**(8)**:** 763-769.

35. Bell ZE, Fristad MA, Youngstrom EA, Arnold LE, Beauchaine TP. Attention-Deficit/Hyperactivity Disorder Symptoms and Externalizing Progression in the LAMS Study: A Test of Trait Impulsivity Theory. *J Am Acad Child Adolesc Psychiatry* 2022; **61**(2)**:** 298-307.

36. Lancaster A, Liljequist L. Cross-validation of PAI scales for the detection of suspected ADHD in adults. *J Clin Psychol* 2018; **74**(10)**:** 1710-1718.

37. Bener A, Kamal M. Predict attention deficit hyperactivity disorder? Evidence -based medicine. *Glob J Health Sci* 2013; **6**(2)**:** 47-57.

38. Lanka P, Rangaprakash D, Gotoor SSR, Dretsch MN, Katz JS, Denney TS *et al.* MALINI (Machine Learning in NeuroImaging): A MATLAB toolbox for aiding clinical diagnostics using resting-state fMRI data. *Data Brief* 2020; **29:** 105213.

39. Bener A, Kamal M, Bener H, Bhugra D. Higher prevalence of iron deficiency as strong predictor of attention deficit hyperactivity disorder in children. *Ann Med Health Sci Res* 2014; **4**(Suppl 3)**:** S291-297.

40. Lanka P, Rangaprakash D, Dretsch MN, Katz JS, Denney TS, Deshpande G. Supervised machine learning for diagnostic classification from large-scale neuroimaging datasets. *Brain Imaging Behav* 2020; **14**(6)**:** 2378-2416.

41. Bennett AE, Power TJ, Eiraldi RB, Leff SS, Blum NJ. Identifying learning problems in children evaluated for ADHD: the Academic Performance Questionnaire. *Pediatrics* 2009; **124**(4)**:** e633-639.

42. Lauth G, Minsel W-R, Koch M. Predictors of success and failure of behavior therapy of ADHD in adults. *Zeitschrift Fur Psychiatrie Psychologie Und Psychotherapie* 2015; **63**(1)**:** 39-46.

43. Berger I, Shenberger IY, Slobodin O. A Machine-Based Prediction Model of ADHD Using CPT Data. *Neurology* 2020; **94**(15).

44. Lee S, Lee W. The association between attention deficit hyperactivity disorder (ADHD) and smoking experience or exposure to environmental tobacco smoke among children and adolescents. *Tob Induc Dis* 2023; **21:** 15.

45. Bergman O, Westberg L, Lichtenstein P, Eriksson E, Larsson H. Study on the possible association of brain-derived neurotrophic factor polymorphism with the developmental course of symptoms of attention deficit and hyperactivity. *Int J Neuropsychopharmacol* 2011; **14**(10)**:** 1367-1376.

46. Li W, Mei X, Wang H, Zhou Y, Huang J, Int Assoc Comp S *et al.* *Link Prediction Boosted Psychiatry Disorder Classification for Functional Connectivity Network*, vol. 102252017.

47. Berloffa S, Salvati A, D'Acunto G, Fantozzi P, Inguaggiato E, Lenzi F *et al.* Internet Gaming Disorder in Children and Adolescents with Attention Deficit Hyperactivity Disorder. *Children (Basel)* 2022; **9**(3).

48. Liang S-F, Hsieh T-H, Chen P-T, Wu M-L, Kung C-C, Lin C-Y *et al.* *Differentiation between Resting-State fMRI data from ADHD and Normal Subjects : Based on Functional Connectivity and Machine Learning*, 2012, 294-298pp.

49. Bianchi L, Espinosa E, Lazzari J, Asnaghi R, Poles I, Clementi L *et al.* Rethinking Theta/Beta Ratio in ADHD through Functional Data Analysis. *Annu Int Conf IEEE Eng Med Biol Soc* 2023; **2023:** 1-4.

50. Liu L, Li W, Su Z, Cook D, Vizioli L, Yacoub E. Efficient estimation via envelope chain in magnetic resonance imaging-based studies. *Scandinavian Journal of Statistics* 2022; **49**(2)**:** 481-501.

51. Brikell I, Wimberley T, Albiñana C, Vilhjálmsson BJ, Agerbo E, Børglum AD *et al.* Interplay of ADHD Polygenic Liability With Birth-Related, Somatic, and Psychosocial Factors in ADHD: A Nationwide Study. *Am J Psychiatry* 2023; **180**(1)**:** 73-88.

52. Liu J, Wang Y-f. Risk factors of attention deficit hyperactivity disorder comorbid with oppositional defiant disorder in children. *Beijing da xue xue bao Yi xue ban = Journal of Peking University Health sciences* 2007; **39**(3)**:** 257-260.

53. Brown MR, Sidhu GS, Greiner R, Asgarian N, Bastani M, Silverstone PH *et al.* ADHD-200 Global Competition: diagnosing ADHD using personal characteristic data can outperform resting state fMRI measurements. *Front Syst Neurosci* 2012; **6:** 69.

54. Liu S, Zhao L, Zhao J, Li B, Wang S-H. Attention deficit/hyperactivity disorder Classification based on deep spatio-temporal features of functional Magnetic Resonance Imaging. *Biomedical Signal Processing and Control* 2022; **71**.

55. Cabana-Domínguez J, Llonga N, Arribas L, Alemany S, Vilar-Ribó L, Demontis D *et al.* Transcriptomic risk scores for attention deficit/hyperactivity disorder. *Mol Psychiatry* 2023; **28**(8)**:** 3493-3502.

56. Liu L, Tang S, Wu F-X, Wang Y-P, Wang J. An Ensemble Hybrid Feature Selection Method for Neuropsychiatric Disorder Classification. *Ieee-Acm Transactions on Computational Biology and Bioinformatics* 2022; **19**(3)**:** 1459-1471.

57. Cahn DA, Marcotte AC, Stern RA, Arruda JE, Akshoomoff NA, Leshko IC. Boston qualitative scoring system for the Rey-Osterrieth Complex Figure: A study of children with attention deficit hyperactivity disorder. *Clinical Neuropsychologist* 1996; **10**(4)**:** 397-406.

58. Liu S, Zhao L, Wang X, Xin Q, Zhao J, Guttery DS *et al.* Deep Spatio-Temporal Representation and Ensemble Classification for Attention Deficit/Hyperactivity Disorder. *Ieee Transactions on Neural Systems and Rehabilitation Engineering* 2021; **29:** 1-10.

59. Cai W, Chen T, Szegletes L, Supekar K, Menon V. Aberrant Cross-Brain Network Interaction in Children With Attention-Deficit/Hyperactivity Disorder and Its Relation to Attention Deficits: A Multisite and Cross-Site Replication Study. *Biol Psychiatry* 2015.

60. Liu L, Feng X, Li H, Li SC, Qian Q, Wang Y. Deep learning model reveals potential risk genes for ADHD, especially Ephrin receptor gene EPHA5. *Briefings in Bioinformatics* 2021; **22**(6).

61. Capusan AJ, Kuja-Halkola R, Bendtsen P, Viding E, McCrory E, Marteinsdottir I *et al.* Childhood maltreatment and attention deficit hyperactivity disorder symptoms in adults: a large twin study. *Psychol Med* 2016; **46**(12)**:** 2637-2646.

62. Liu Y, Qu H-Q, Chang X, Nguyen K, Qu J, Tian L *et al.* Deep learning prediction of attention-deficit hyperactivity disorder in African Americans by copy number variation. *Experimental Biology and Medicine* 2021; **246**(21)**:** 2317-2323.

63. Catherine Joy R, Thomas George S, Albert Rajan A, Subathra MSP. Detection of ADHD From EEG Signals Using Different Entropy Measures and ANN. *Clin EEG Neurosci* 2022; **53**(1)**:** 12-23.

64. Liu Y, Qu H-Q, Mentch FD, Qu J, Chang X, Nguyen K *et al.* Application of deep learning algorithm on whole genome sequencing data uncovers structural variants associated with multiple mental disorders in African American patients. *Molecular Psychiatry* 2022; **27**(3)**:** 1469-1478.

65. Cervantes-Henríquez ML, Acosta-López JE, Martinez AF, Arcos-Burgos M, Puentes-Rozo PJ, Vélez JI. Machine Learning Prediction of ADHD Severity: Association and Linkage to. *J Atten Disord* 2022; **26**(4)**:** 587-605.

66. Loftness BC, Rizzo DM, Halvorson-Phelan J, O'Leary A, Prytherch S, Bradshaw C *et al.* Toward Digital Phenotypes of Early Childhood Mental Health via Unsupervised and Supervised Machine Learning. *Annu Int Conf IEEE Eng Med Biol Soc* 2023; **2023:** 1-4.

67. Cervantes-Henríquez ML, Acosta-López JE, Martínez-Banfi ML, Vélez JI, Mejía-Segura E, Lozano-Gutiérrez SG *et al.* ADHD Endophenotypes in Caribbean Families. *J Atten Disord* 2020; **24**(14)**:** 2100-2114.

68. Loney J, Prinz RJ, Mishalow J, Joad J. Hyperkinetic/aggressive boys in treatment: predictors of clinical response to methylphenidate. *Am J Psychiatry* 1978; **135**(12)**:** 1487-1491.

69. An Approach to Measure and Improve the Cognitive Capability of ADHD Affected Children through EEG Signals. *Proceedings of the 18th IEEE International Conference on Advanced Learning Technologies (ICALT)*; 2018

Jul 09-13 2018; Indian Inst Technol Bombay, Bombay, INDIA2018.

70. Antonio Lopez-Villalobos J, Maria Andres-de Llano J, Delgado Sanchez-Mateos J, Rodriguez-Molinero L, Garrido-Redondo M, Teresa Martinez-Rivera M *et al.* Validity of a reduced model of items of DSM-IV criteria according to the parents' and teachers' responses in the diagnostic of the Attention Deficit Hyperactivity Disorder (Combined Type). *Anales De Psicologia* 2014; **30**(3)**:** 898-907.

71. Chang J-C, Lin H-Y, Lv J, Tseng W-YI, Gau SS-F. Regional brain volume predicts response to methylphenidate treatment in individuals with ADHD. *Bmc Psychiatry* 2021; **21**(1).

72. Antonio Lopez-Villalobos J, Serrano-Pintado I, Delgado-Sanchez-Mateos J, Maria Andres de Llano J, Alberola-Lopez S, Maria Sacristan-Martin A *et al.* Use of Matching Familiar Figures Test 20 in the diagnosis of children with Attention Deficit Hyperactivity Disorder. *International Journal of Clinical and Health Psychology* 2010; **10**(3)**:** 499-517.

73. Chang Y, Stevenson C, Chen IC, Lin D-S, Ko L-W. Neurological state changes indicative of ADHD in children learned via EEG-based LSTM networks. *Journal of Neural Engineering* 2022; **19**(1).

74. Antonio Lopez-Villalobos J, Serrano-Pintado I, Maria Andres-De Llano J, Delgado Sanchez-Mateos J, Alberola-Lopez S, Isabel Sanchez-Azon M. Usefulness of the Stroop test in attention deficit hyperactivity disorder. *Revista De Neurologia* 2010; **50**(6)**:** 333-340.

75. Chang-Hyun R, Hwang Y. Artificial Intelligence Analysis of Bio-signals for Automated Detection and Automated Diagnosis of ADHD and CD. *Korean Journal of Correctional Discourse* 2021; **15**(3)**:** 87-118.

76. Lopez Villalobos JA, Serrano Pintado I, Delgado Sanchez-Mateos J, Andres de Llano JM, Sanchez Azon MI, Alberola Lopez S. Utility of a statistical model of cognitive styles in attention deficit hyperactivity disorder. *Psicothema* 2011; **23**(4)**:** 818-823.

77. Charach A, Lin E, To T. Evaluating the Hyperactivity/Inattention Subscale of the National Longitudinal Survey of Children and Youth. *Health Rep* 2010; **21**(2)**:** 43-50.

78. López-Villalobos JA, Serrano-Pintado I, Andrés-De Llano JM, Sánchez-Mateos JD, Alberola-López S, Sánchez-Azón MI. [Usefulness of the Stroop test in attention deficit hyperactivity disorder]. *Rev Neurol* 2010; **50**(6)**:** 333-340.

79. Engagement Analysis of ADHD Students using Visual Cues from Eye Tracker. *Proceedings of the International Conference on Multimodal Interaction (ICMI)*; 2020

Oct 25-29 2020; Electr Network2020.

80. Luo Y, Alvarez TL, Halperin JM, Li X. Multimodal neuroimaging-based prediction of adult outcomes in childhood-onset ADHD using ensemble learning techniques. *Neuroimage Clin* 2020; **26:** 102238.

81. Chauhan N, Choi B-J. DNN based Classification of ADHD fMRI Data using Functional Connectivity Coefficient. *International Journal of Fuzzy Logic and Intelligent Systems* 2020; **20**(4)**:** 255-260.

82. Mafi M, Radfar S. High Dimensional Convolutional Neural Network for EEG Connectivity-Based Diagnosis of ADHD. *J Biomed Phys Eng* 2022; **12**(6)**:** 645-654.

83. Chen H, Chen W, Song Y, Sun L, Li X. EEG characteristics of children with attention-deficit/hyperactivity disorder. *Neuroscience* 2019; **406:** 444-456.

84. Magee CA, Clarke AR, Barry RJ, McCarthy R, Selikowitz M. Examining the diagnostic utility of EEG power measures in children with attention deficit/hyperactivity disorder. *Clin Neurophysiol* 2005; **116**(5)**:** 1033-1040.

85. Chen H, Song Y, Li X. Use of deep learning to detect personalized spatial-frequency abnormalities in EEGs of children with ADHD. *J Neural Eng* 2019; **16**(6)**:** 066046.

86. Malegiannaki AC, Aretouli E, Metallidou P, Messinis L, Zafeiriou D, Kosmidis MH. Test of Everyday Attention for Children (TEA-Ch): Greek Normative Data and Discriminative Validity for Children with Combined Type of Attention Deficit-Hyperactivity Disorder. *Dev Neuropsychol* 2019; **44**(2)**:** 189-202.

87. Chen Y, Tang Y, Wang C, Liu X, Zhao L, Wang Z. ADHD classification by dual subspace learning using resting-state functional connectivity. *Artif Intell Med* 2020; **103:** 101786.

88. Mancera Valetts LP, Baldiris Navarro SM, Betancur Chicué V. Indicators of ADHD symptoms in virtual learning context using machine learning technics. *Revista EAN* 2015; (79)**:** 22-37.

89. Cheng C-Y, Tseng W-L, Chang C-F, Chang C-H, Gau SS-F. A Deep Learning Approach for Missing Data Imputation of Rating Scales Assessing Attention-Deficit Hyperactivity Disorder. *Frontiers in Psychiatry* 2020; **11**.

90. Moura O, Pereira M, Alfaiate C, Fernandes E, Fernandes B, Nogueira S *et al.* Neurocognitive functioning in children with developmental dyslexia and attention-deficit/hyperactivity disorder: Multiple deficits and diagnostic accuracy. *J Clin Exp Neuropsychol* 2017; **39**(3)**:** 296-312.

91. Cheng W, Ji X, Zhang J, Feng J. Individual classification of ADHD patients by integrating multiscale neuroimaging markers and advanced pattern recognition techniques. *Front Syst Neurosci* 2012; **6:** 58.

92. Mueller A, Candrian G, Kropotov JD, Ponomarev VA, Baschera GM. Classification of ADHD patients on the basis of independent ERP components using a machine learning system. *Nonlinear Biomed Phys* 2010; **4:** S1.

93. Cho S, Shin MS. Neural network based automatic diagnosis of children with brain dysfunction. *Int J Neural Syst* 2001; **11**(4)**:** 361-369.

94. Munkvold LH, Manger T, Lundervold AJ. Conners' continuous performance test (CCPT-II) in children with ADHD, ODD, or a combined ADHD/ODD diagnosis. *Child Neuropsychol* 2014; **20**(1)**:** 106-126.

95. Detection of Attention Deficit Hyperactivity Disorder Using Local and Global Features. *Proceedings of the Medical Technologies National Congress (TIPTEKNO)*; 2018

Nov 08-10 2018; Magusa, CYPRUS2018.

96. Murugesan GK, Ganesh C, Nalawade S, Davenport EM, Wagner B, Kim WH *et al.* BrainNET: Inference of Brain Network Topology Using Machine Learning. *Brain Connect* 2020; **10**(8)**:** 422-435.

97. Classification of ADHD Using Ensemble Algorithms with Deep Learning and Hand Crafted Features. *Proceedings of the Medical Technologies Congress (TIPTEKNO)*; 2019

Oct 03-05 2019; Izmir, TURKEY2019.

98. Nagai T, Kurihara T, Koya H, Nakano Y, Sugisawa S, Sambe T *et al.* Identification of factors associated with the efficacy of atomoxetine in adult attention-deficit/hyperactivity disorder. *Neuropsychopharmacol Rep* 2022; **42**(3)**:** 249-255.

99. The Effect of Data Augmentation on ADHD Diagnostic Model using Deep Learning. *Proceedings of the Medical Technologies Congress (TIPTEKNO)*; 2019

Oct 03-05 2019; Izmir, TURKEY2019.

100. Østergaard SD, Larsen JT, Dalsgaard S, Wilens TE, Mortensen PB, Agerbo E *et al.* Predicting ADHD by Assessment of Rutter's Indicators of Adversity in Infancy. *PLoS One* 2016; **11**(6)**:** e0157352.

101. Resting State EEG Classification of Children With ADHD. *Proceedings of the IEEE Signal Processing in Medicine and Biology Symposium*; 2020

Dec 05 2020; Philadelphia, PA2020.

102. Overgaard KR, Madsen KB, Oerbeck B, Friis S, Obel C. The predictive validity of the Strengths and Difficulties Questionnaire for child attention-deficit/hyperactivity disorder. *Eur Child Adolesc Psychiatry* 2019; **28**(5)**:** 625-633.

103. Colby JB, Rudie JD, Brown JA, Douglas PK, Cohen MS, Shehzad Z. Insights into multimodal imaging classification of ADHD. *Front Syst Neurosci* 2012; **6:** 59.

104. Overgaard KR, Oerbeck B, Friis S, Biele G, Pripp AH, Aase H *et al.* Screening with an ADHD-specific rating scale in preschoolers: A cross-cultural comparison of the Early Childhood Inventory-4. *Psychol Assess* 2019; **31**(8)**:** 985-994.

105. Cordova M, Shada K, Demeter DV, Doyle O, Miranda-Dominguez O, Perrone A *et al.* Heterogeneity of executive function revealed by a functional random forest approach across ADHD and ASD. *Neuroimage Clin* 2020; **26:** 102245.

106. Machine-Based Learning System: Classification of ADHD and non-ADHD participants. *Proceedings of the 25th Signal Processing and Communications Applications Conference (SIU)*; 2017

May 15-18 2017; Antalya, TURKEY2017.

107. Curtin P, Neufeld J, Curtin A, Arora M, Bölte S. Altered Periodic Dynamics in the Default Mode Network in Autism and Attention-Deficit/Hyperactivity Disorder. *Biol Psychiatry* 2022; **91**(11)**:** 956-966.

108. Parashar A, Kalra N, Singh J, Goyal RK. Machine Learning Based Framework for Classification of Children with ADHD and Healthy Controls. *Intelligent Automation and Soft Computing* 2021; **28**(3)**:** 669-682.

109. Das W, Khanna S. A Robust Machine Learning Based Framework for the Automated Detection of ADHD Using Pupillometric Biomarkers and Time Series Analysis. *Sci Rep* 2021; **11**(1)**:** 16370.

110. Park C, Rouzi MD, Atique MMU, Finco MG, Mishra RK, Barba-Villalobos G *et al.* Machine Learning-Based Aggression Detection in Children with ADHD Using Sensor-Based Physical Activity Monitoring. *Sensors (Basel)* 2023; **23**(10).

111. Davis AS, Finch WH, Boseck JJ, Dean RS. Classification and regression tree analysis of cognitive processing abilities in the differentiation of ADHD subtypes. *Archives of Clinical Neuropsychology* 2008; **23**(6)**:** 716-716.

112. Interpretable Diagnosis of ADHD Based on Wavelet Features and Logistic Regression. *Proceedings of the 7th International Workshop on Artificial Intelligence and Pattern Recognition (IWAIPR)*; 2021

Oct 05-07 2021; Electr Network2021.

113. A Big-Data-Analytics Framework for Supporting Classification of ADHD and Healthy Children via Principal Component Analysis of EEG Sleep Spindles Power Spectra. *Proceedings of the 23rd KES International Conference on Knowledge-Based and Intelligent Information and Engineering Systems (KES)*; 2019

Sep 04-06 2019; Budapest, HUNGARY2019.

114. Paucke M, Stark T, Exner C, Kallweit C, Hegerl U, Strauß M. [Attention deficit-hyperactivity disorder (ADHD) and comorbid mental disorders : ADHD-specific self-rating scales in differential diagnostics]. *Nervenarzt* 2018; **89**(11)**:** 1287-1293.

115. De Silva S, Dayarathna S, Ariyarathne G, Meedeniya D, Jayarathna S, Michalek AMP. Computational Decision Support System for ADHD Identification. *International Journal of Automation and Computing* 2021; **18**(2)**:** 233-255.

116. Peng X, Lin P, Zhang T, Wang J. Extreme learning machine-based classification of ADHD using brain structural MRI data. *PLoS One* 2013; **8**(11)**:** e79476.

117. De Silva S, Dayarathna S, Ariyarathne G, Meedeniya D, Jayarathna S. A Survey of Attention Deficit Hyperactivity Disorder Identification Using Psychophysiological Data. *International Journal of Online and Biomedical Engineering* 2019; **15**(13)**:** 61-76.

118. Pettersson R, Söderström S, Nilsson KW. Diagnosing ADHD in Adults: An Examination of the Discriminative Validity of Neuropsychological Tests and Diagnostic Assessment Instruments. *J Atten Disord* 2018; **22**(11)**:** 1019-1031.

119. Dimitrov LV, Kaminski JW, Holbrook JR, Bitsko RH, Yeh M, Courtney JG *et al.* A Systematic Review and Meta-analysis of Chemical Exposures and Attention-Deficit/Hyperactivity Disorder in Children. *Prev Sci* 2023.

120. Power TJ, Andrews TJ, Eiraldi RB, Doherty BJ, Ikeda MJ, DuPaul G *et al.* Evaluating attention deficit hyperactivity disorder using multiple informants: The incremental utility of combining teacher with parent reports. *Psychological Assessment* 1998; **10**(3)**:** 250-260.

121. Dincer ED, Bakar EE, Taner YI, Soysal AS, Turgay A, Karakas S. The Relation of Conners Rating Scale with Executive Functions. *Turkiye Klinikleri Tip Bilimleri Dergisi* 2012; **32**(4)**:** 1011-1025.

122. Power TJ, Doherty BJ, Panichelli-Mindel SM, Karustis JL, Eiraldi RB, Anastopoulos AD *et al.* The predictive validity of parent and teacher reports of ADHD symptoms. *Journal of Psychopathology and Behavioral Assessment* 1998; **20**(1)**:** 57-81.

123. Dou C, Zhang S, Wang H, Sun L, Huang Y, Yue W. ADHD fMRI short-time analysis method for edge computing based on multi-instance learning. *Journal of Systems Architecture* 2020; **111**.

124. Predescu E, Sipos R, Dobrean A, Miclutia I. THE DISCRIMINATIVE POWER OF THE CBCL 1.5-5 BETWEEN AUTISM SPECTRUM DISORDERS AND OTHER PSYCHIATRIC DISORDERS. *Journal of Cognitive and Behavioral Psychotherapies* 2013; **13**(1)**:** 75-87.

125. Du J, Wang L, Jie B, Zhang D. Network-based classification of ADHD patients using discriminative subnetwork selection and graph kernel PCA. *Comput Med Imaging Graph* 2016; **52:** 82-88.

126. Qureshi MN, Min B, Jo HJ, Lee B. Multiclass Classification for the Differential Diagnosis on the ADHD Subtypes Using Recursive Feature Elimination and Hierarchical Extreme Learning Machine: Structural MRI Study. *PLoS One* 2016; **11**(8)**:** e0160697.

127. Dubreuil-Vall L, Ruffini G, Camprodon JA. Deep Learning Convolutional Neural Networks Discriminate Adult ADHD From Healthy Individuals on the Basis of Event-Related Spectral EEG. *Front Neurosci* 2020; **14:** 251.

128. ADHD SUBGROUP DISCRIMINATION WITH GLOBAL CONNECTIVITY FEATURES USING HIERARCHICAL EXTREME LEARNING MACHINE: RESTING-STATE FMRI STUDY. *Proceedings of the IEEE 14th International Symposium on Biomedical Imaging (ISBI) - From Nano to Macro*; 2017

Apr 18-21 2017; Melbourne, AUSTRALIA2017.

129. Duda M, Haber N, Daniels J, Wall DP. Crowdsourced validation of a machine-learning classification system for autism and ADHD. *Transl Psychiatry* 2017; **7**(5)**:** e1133.

130. Qureshi MNI, Oh J, Min B, Jo HJ, Lee B. Multi-modal, Multi-measure, and Multi-class Discrimination of ADHD with Hierarchical Feature Extraction and Extreme Learning Machine Using Structural and Functional Brain MRI. *Front Hum Neurosci* 2017; **11:** 157.

131. Duda M, Ma R, Haber N, Wall DP. Use of machine learning for behavioral distinction of autism and ADHD. *Transl Psychiatry* 2016; **6**(2)**:** e732.

132. Rezaei M, Zare H, Hakimdavoodi H, Nasseri S, Hebrani P. Classification of drug-naive children with attention-deficit/hyperactivity disorder from typical development controls using resting-state fMRI and graph theoretical approach. *Front Hum Neurosci* 2022; **16:** 948706.

133. Efe A, Kaba D, Canlı M, Temeltürk RD. Impact of Attention-Deficit/Hyperactivity Disorder Comorbidity on Phenomenology and Treatment Outcomes of Pediatric Obsessive-Compulsive Disorder. *J Child Adolesc Psychopharmacol* 2022; **32**(6)**:** 337-348.

134. Riaz A, Asad M, Alonso E, Slabaugh G. Fusion of fMRI and non-imaging data for ADHD classification. *Comput Med Imaging Graph* 2018; **65:** 115-128.

135. Eloyan A, Muschelli J, Nebel MB, Liu H, Han F, Zhao T *et al.* Automated diagnoses of attention deficit hyperactive disorder using magnetic resonance imaging. *Front Syst Neurosci* 2012; **6:** 61.

136. Richarte V, Corrales M, Pozuelo M, Serra-Pla J, Ibanez P, Calvo E *et al.* Spanish validation of the adult Attention Deficit/Hyperactivity Disorder Rating Scale (ADHD-RS): relevance of clinical subtypes. *Revista De Psiquiatria Y Salud Mental* 2017; **10**(4)**:** 185-191.

137. Eslami T, Almuqhim F, Raiker JS, Saeed F. Machine Learning Methods for Diagnosing Autism Spectrum Disorder and Attention- Deficit/Hyperactivity Disorder Using Functional and Structural MRI: A Survey. *Front Neuroinform* 2020; **14:** 575999.

138. Rimvall MK, Elberling H, Rask CU, Helenius D, Skovgaard AM, Jeppesen P. Predicting ADHD in school age when using the Strengths and Difficulties Questionnaire in preschool age: a longitudinal general population study, CCC2000. *Eur Child Adolesc Psychiatry* 2014; **23**(11)**:** 1051-1060.

139. Evans SC, Cooley JL, Blossom JB, Pederson CA, Tampke EC, Fite PJ. Examining ODD/ADHD Symptom Dimensions as Predictors of Social, Emotional, and Academic Trajectories in Middle Childhood. *J Clin Child Adolesc Psychol* 2020; **49**(6)**:** 912-929.

140. Rishel CW, Greeno C, Marcus SC, Shear MK, Anderson C. Use of the child behavior checklist as a diagnostic screening tool in community mental health. *Research on Social Work Practice* 2005; **15**(3)**:** 195-203.

141. Faraone SV. IMPROVING MACHINE LEARNING PREDICTION OF ADHD USING GENE SET POLYGENIC RISK SCORES AND RISK SCORES FROM GENETICALLY CORRELATED DISORDERS. *Journal of the American Academy of Child and Adolescent Psychiatry* 2021; **60**(10)**:** S303-S303.

142. Sabuncu MR, Konukoglu E, Alzheimer's Dis N. Clinical Prediction from Structural Brain MRI Scans: A Large-Scale Empirical Study. *Neuroinformatics* 2015; **13**(1)**:** 31-46.

143. Investigation of Low Frequency Drift in Attention Deficit Hyperactivity Disorder fMRI Signal. *Proceedings of the International Conference on Information Science and Technology (ICIST)*; 2013

Mar 23-25 2013; Yangzhou, PEOPLES R CHINA2013.

144. Sachnev V, Suresh S, Sundararajan N, Mahanand BS, Azeem MW, Saraswathi S. Multi-Region Risk-Sensitive Cognitive Ensembler for Accurate Detection of Attention-Deficit/Hyperactivity Disorder. *Cognitive Computation* 2019; **11**(4)**:** 545-559.

145. Fuermaier ABM, Tucha O, Koerts J, Lange KW, Weisbrod M, Aschenbrenner S *et al.* Noncredible cognitive performance at clinical evaluation of adult ADHD: An embedded validity indicator in a visuospatial working memory test. *Psychol Assess* 2017; **29**(12)**:** 1466-1479.

146. Sadatnezhad K, Boostani R, Ghanizadeh A. Proposing an adaptive mutation to improve XCSF performance to classify ADHD and BMD patients. *J Neural Eng* 2010; **7**(6)**:** 066006.

147. Fuermaier AB, Tucha O, Koerts J, Grabski M, Lange KW, Weisbrod M *et al.* The Development of an Embedded Figures Test for the Detection of Feigned Attention Deficit Hyperactivity Disorder in Adulthood. *PLoS One* 2016; **11**(10)**:** e0164297.

148. Shin H, Yuniar CT, Oh S, Purja S, Park S, Lee H *et al.* The Adverse Effects and Nonmedical Use of Methylphenidate Before and After the Outbreak of COVID-19: Machine Learning Analysis. *J Med Internet Res* 2023; **25:** e45146.

149. Identification of ADHD Cognitive Pattern Disturbances Using EEG and Wavelets Analysis. *Proceedings of the 17th IEEE International Conference on Bioinformatics and Bioengineering (BIBE)*; 2017

Oct 23-25 2017; Herndon, VA2017.

150. Shao L, Xu Y, Fu D. Classification of ADHD with bi-objective optimization. *J Biomed Inform* 2018; **84:** 164-170.

151. Gao X-P, Su L-Y, Zhao A-L, Luo X-R, Xia K. Association of 14 polymorphisms in the five candidate genes and attention deficit hyperactivity disorder. *Zhongguo dang dai er ke za zhi = Chinese journal of contemporary pediatrics* 2009; **11**(8)**:** 617-622.

152. Shao L, You Y, Du H, Fu D. Classification of ADHD with fMRI data and multi-objective optimization. *Comput Methods Programs Biomed* 2020; **196:** 105676.

153. Learning a Phenotypic-Attribute Attentional Brain Connectivity Embedding for ADHD Classification using rs-fMRI. *Proceedings of the 42nd Annual International Conference of the IEEE-Engineering-in-Medicine-and-Biology-Society (EMBC)*; 2020

Jul 20-24 2020; Montreal, CANADA2020.

154. Shao L, Zhang D, Du H, Fu D. Deep Forest in ADHD Data Classification. *Ieee Access* 2019; **7:** 137913-137919.

155. Gehricke J-G, Kruggel F, Thampipop T, Alejo SD, Tatos E, Fallon J *et al.* The brain anatomy of attention-deficit/hyperactivity disorder in young adults - a magnetic resonance imaging study. *Plos One* 2017; **12**(4).

156. Sharma V, Kim J-W, Ryan N. Predicting Side Effects of Methylphenidate in ADHD : A Machine Learning Approach. *Biological Psychiatry* 2014; **75**(9)**:** 122S-123S.

157. Gémes K, Taipale H, Björkenstam E, Rahman S, Gustafsson K, Tanskanen A *et al.* The role of sociodemographic and clinical factors in the initiation and discontinuation of attention deficit hyperactivity disorder medication among young adults in Sweden. *Front Psychiatry* 2023; **14:** 1152286.

158. ADHD Children Identification Based on EEG Using Effective Connectivity Techniques. *Proceedings of the 10th International Conference on Health Information Science (HIS)*; 2021

Oct 25-28 2021; Melbourne, AUSTRALIA2021.

159. Ghaderyan P, Moghaddam F, Khoshnoud S, Shamsi M. New interdependence feature of EEG signals as a biomarker of timing deficits evaluated in Attention-Deficit/Hyperactivity Disorder detection. *Measurement* 2022; **199**.

160. Shepler DK, Callan PD. Differences in executive functioning between adults with ADHD and those diagnosed with other psychiatric diagnoses: Utility of the CTMT and the WAIS-IV. *Appl Neuropsychol Adult* 2022**:** 1-10.

161. Ghasemi E, Ebrahimi M, Ebrahimie E. Machine learning models effectively distinguish attention-deficit/ hyperactivity disorder using event-related potentials. *Cognitive Neurodynamics* 2022.

162. Sheriff M, Gayathri R. An enhanced ensemble machine learning classification method to detect attention deficit hyperactivity for various artificial intelligence and telecommunication applications. *Computational Intelligence* 2022.

163. Goh PK, Martel MM, Jones PJ, Bansal PS, Eng AG, Elkins AR *et al.* Clarifying Relations Between ADHD and Functional Impairment in Adulthood: Utilization of Network and Machine Learning Approaches. *Assessment* 2021**:** 10731911211050921.

164. ADHD Classification Using Bag of Words Approach on Network Features. *Proceedings of the Conference on Medical Imaging - Image Processing*; 2012

Feb 06-09 2012; San Diego, CA2012.

165. Gokten ES, Tulay EE, Beser B, Yuksel ME, Arikan K, Tarhan N *et al.* Predictive Value of Slow and Fast EEG Oscillations for Methylphenidate Response in ADHD. *Clinical Eeg and Neuroscience* 2019; **50**(5)**:** 332-338.

166. Son HM, Lee DG, Joung Y-S, Lee JW, Seok EJ, Chung T-M *et al.* A novel approach to diagnose ADHD using virtual reality. *International Journal of Web Information Systems* 2021; **17**(5)**:** 516-536.

167. Grunblatt E, Geissler J, Jacob CP, Renner T, Muller M, Bartl J *et al.* Pilot study: potential transcription markers for adult attention-deficit hyperactivity disorder in whole blood. *Attention deficit and hyperactivity disorders* 2012; **4**(2)**:** 77-84.

168. Sudre G, Sharp W, Kundzicz P, Bouyssi-Kobar M, Norman L, Choudhury S *et al.* Predicting the course of ADHD symptoms through the integration of childhood genomic, neural, and cognitive features. *Mol Psychiatry* 2021; **26**(8)**:** 4046-4054.

169. Gu Y, Miao S, Yang J, Li X. ADHD Children Identification With Multiview Feature Fusion of fNIRS Signals. *Ieee Sensors Journal* 2022; **22**(13)**:** 13536-13543.

170. Tan L, Guo X, Ren S, Epstein JN, Lu LJ. A Computational Model for the Automatic Diagnosis of Attention Deficit Hyperactivity Disorder Based on Functional Brain Volume. *Front Comput Neurosci* 2017; **11:** 75.

171. Guney G, Kisacik E, Kalaycioglu C, Saygili G. Exploring the attention process differentiation of attention deficit hyperactivity disorder (ADHD) symptomatic adults using artificial intelligence on electroencephalography (EEG) signals. *Turkish Journal of Electrical Engineering and Computer Sciences* 2021; **29**(5)**:** 2312-2325.

172. Ter-Minassian L, Viani N, Wickersham A, Cross L, Stewart R, Velupillai S *et al.* Assessing machine learning for fair prediction of ADHD in school pupils using a retrospective cohort study of linked education and healthcare data. *BMJ Open* 2022; **12**(12)**:** e058058.

173. Guntuku SC, Ramsay JR, Merchant RM, Ungar LH. Language of ADHD in Adults on Social Media. *J Atten Disord* 2019; **23**(12)**:** 1475-1485.

174. Thorell LB, Sjöwall D, Mies GW, Scheres A. Quick Delay Questionnaire: Reliability, validity, and relations to functional impairments in adults with attention-deficit/hyperactivity disorder (ADHD). *Psychol Assess* 2017; **29**(10)**:** 1261-1272.

175. Guo Y, Wang J, Yan S, Sui S. Clinical Efficacy Evaluation of Psychological Nursing Intervention Combined with Drugs Treatment of Children with ADHD under Artificial Intelligence. *J Healthc Eng* 2022; **2022:** 1818693.

176. Tran T, Kavuluru R. Predicting mental conditions based on "history of present illness" in psychiatric notes with deep neural networks. *J Biomed Inform* 2017; **75s:** S138-s148.

177. Gupta R, Kar BR, Srinivasan N. Cognitive-motivational deficits in ADHD: development of a classification system. *Child Neuropsychol* 2011; **17**(1)**:** 67-81.

178. Trognon A, Richard M. Questionnaire-based computational screening of adult ADHD. *BMC Psychiatry* 2022; **22**(1)**:** 401.

179. Gurevitz M, Geva R, Varon M, Leitner Y. Early markers in infants and toddlers for development of ADHD. *J Atten Disord* 2014; **18**(1)**:** 14-22.

180. Uchida M, Bukhari Q, DiSalvo M, Green A, Serra G, Hutt Vater C *et al.* Can machine learning identify childhood characteristics that predict future development of bipolar disorder a decade later? *J Psychiatr Res* 2022; **156:** 261-267.

181. Hadas I, Hadar A, Lazarovits A, Daskalakis ZJ, Zangen A. Right prefrontal activation predicts ADHD and its severity: A TMS-EEG study in young adults. *Prog Neuropsychopharmacol Biol Psychiatry* 2021; **111:** 110340.

182. ADHD and ASD Classification Based on Emotion Recognition Data. *Proceedings of the 15th IEEE International Conference on Machine Learning and Applications (ICMLA)*; 2016

Dec 18-20 2016; Anaheim, CA2016.

183. Handen BL, Janosky J, McAuliffe S, Breaux AM, Feldman H. Prediction of response to methylphenidate among children with ADHD and mental retardation. *J Am Acad Child Adolesc Psychiatry* 1994; **33**(8)**:** 1185-1193.

184. Ustun B, Adler LA, Rudin C, Faraone SV, Spencer TJ, Berglund P *et al.* The World Health Organization Adult Attention-Deficit/Hyperactivity Disorder Self-Report Screening Scale for DSM-5. *JAMA Psychiatry* 2017; **74**(5)**:** 520-527.

185. Hart H, Chantiluke K, Cubillo AI, Smith AB, Simmons A, Brammer MJ *et al.* Pattern classification of response inhibition in ADHD: toward the development of neurobiological markers for ADHD. *Hum Brain Mapp* 2014; **35**(7)**:** 3083-3094.

186. van der Meer D. Predicting attention-deficit/hyperactivity disorder severity from psychosocial stress and stress response genes: a random forest regression approach. *Behavior Genetics* 2017; **47**(6)**:** 646-646.

187. Hazell PL, Lewin TJ, McDowell MJ, Walton JM. Factors associated with medium-term response to psychostimulant medication. *J Paediatr Child Health* 1999; **35**(3)**:** 264-268.

188. Wang AM, Pradhan S, Coughlin JM, Trivedi A, DuBois SL, Crawford JL *et al.* Assessing Brain Metabolism With 7-T Proton Magnetic Resonance Spectroscopy in Patients With First-Episode Psychosis. *JAMA Psychiatry* 2019; **76**(3)**:** 314-323.

189. Heller MD, Roots K, Srivastava S, Schumann J, Srivastava J, Hale TS. A Machine Learning-Based Analysis of Game Data for Attention Deficit Hyperactivity Disorder Assessment. *Games Health J* 2013; **2**(5)**:** 291-298.

190. Wang X-H, Xu J, Li L. Estimating individual scores of inattention and impulsivity based on dynamic features of intrinsic connectivity network. *Neuroscience Letters* 2020; **724**.

191. Hill BD, Pella RD, Singh AN, Jones GN, Gouvier WD. The Wender Utah Rating Scale: Adult ADHD diagnostic tool or personality index? *J Atten Disord* 2009; **13**(1)**:** 87-94.

192. Wang X-H, Jiao Y, Li L. PREDICTING CLINICAL SYMPTOMS OF ATTENTION DEFICIT HYPERACTIVITY DISORDER BASED ON TEMPORAL PATTERNS BETWEEN AND WITHIN INTRINSIC CONNECTIVITY NETWORKS. *Neuroscience* 2017; **362:** 60-69.

193. Hirvikoski T, Lajic S, Jokinen J, Renhorn E, Trillingsgaard A, Kadesjö B *et al.* Using the five to fifteen-collateral informant questionnaire for retrospective assessment of childhood symptoms in adults with and without autism or ADHD. *Eur Child Adolesc Psychiatry* 2021; **30**(9)**:** 1367-1381.

194. Wang C, Wang X, Jing X, Yokoi H, Huang W, Zhu M *et al.* Towards high-accuracy classifying attention-deficit/hyperactivity disorders using CNN-LSTM model. *J Neural Eng* 2022; **19**(4).

195. Hu L, Wan Q, Huang L, Tang J, Huang S, Chen X *et al.* MRI-based brain age prediction model for children under 3 years old using deep residual network. *Brain Struct Funct* 2023; **228**(7)**:** 1771-1784.

196. Wang D, Hong D, Wu Q. Attention Deficit Hyperactivity Disorder Classification Based on Deep Learning. *IEEE/ACM Trans Comput Biol Bioinform* 2023; **20**(2)**:** 1581-1586.

197. Huang X-X, Ou P, Qian Q-F, Huang Y, Wang Y-X. A prospective study of the decision tree prediction model for attention deficit hyperactivity disorder in preschool children. *Zhongguo dang dai er ke za zhi = Chinese journal of contemporary pediatrics* 2022; **24**(3)**:** 255-260.

198. Wang G, Li W, Huang S, Chen Z. A Prospective Study of an Early Prediction Model of Attention Deficit Hyperactivity Disorder Based on Artificial Intelligence. *J Atten Disord* 2023**:** 10870547231211360.

199. Iannaccone R, Hauser TU, Ball J, Brandeis D, Walitza S, Brem S. Classifying adolescent attention-deficit/hyperactivity disorder (ADHD) based on functional and structural imaging. *European Child & Adolescent Psychiatry* 2015; **24**(10)**:** 1279-1289.

200. Wiguna T, Bahana R, Dirgantoro B, Minayati K, Teh SD, Ismail RI *et al.* Developing attention deficits/hyperactivity disorder-virtual reality diagnostic tool with machine learning for children and adolescents. *Front Psychiatry* 2022; **13:** 984481.

201. Igual L, Carles Soliva J, Gimeno R, Escalera S, Vilarroya O, Radeva P *et al.* *Automatic Internal Segmentation of Caudate Nucleus for Diagnosis of Attention-Deficit/Hyperactivity Disorder*, vol. 73252012, 222-229pp.

202. Yao D, Guo X, Zhao Q, Liu L, Cao Q, Wang Y *et al.* Discriminating ADHD From Healthy Controls Using a Novel Feature Selection Method Based on Relative Importance and Ensemble Learning. *Annu Int Conf IEEE Eng Med Biol Soc* 2018; **2018:** 4632-4635.

203. Ishizuya A, Enomoto M, Tachimori H, Takahashi H, Sugihara G, Kitamura S *et al.* Risk factors for low adherence to methylphenidate treatment in pediatric patients with attention-deficit/hyperactivity disorder. *Sci Rep* 2021; **11**(1)**:** 1707.

204. Zhang D, Zhang R, Zhou L, Zhou K, Chang C. The brain network underlying attentional blink predicts symptoms of attention deficit hyperactivity disorder in children. *Cereb Cortex* 2023; **33**(6)**:** 2761-2773.

205. Jafari P, Mehrabani-Zeinabad K, Javadi S, Ghanizadeh A, Bagheri Z. A Machine Learning Approach to Assess Differential Item Functioning of the KINDL Quality of Life Questionnaire Across Children with and Without ADHD. *Child Psychiatry Hum Dev* 2022; **53**(5)**:** 980-991.

206. Zhu F, Liu B, Kuang D, Zhu X, Bi X, Song Y *et al.* The association between physical activity and sleep in adult ADHD patients with stimulant medication use. *Front Psychiatry* 2023; **14:** 1236636.

207. Jansen AG, Jansen PR, Savage JE, Kraft J, Skarabis N, Polderman TJC *et al.* The predictive capacity of psychiatric and psychological polygenic risk scores for distinguishing cases in a child and adolescent psychiatric sample from controls. *J Child Psychol Psychiatry* 2021; **62**(9)**:** 1079-1089.

208. Abibullaev B, An J. Decision support algorithm for diagnosis of ADHD using electroencephalograms. *J Med Syst* 2012; **36**(4)**:** 2675-2688.

209. Acosta-Lopez JE, Suarez I, Pineda DA, Cervantes-Henriquez ML, Martinez-Banfi ML, Lozano-Gutierrez SG *et al.* Impulsive and Omission Errors: Potential Temporal Processing Endophenotypes in ADHD. *Brain Sciences* 2021; **11**(9).

210. Ahire N, Awale RN, Wagh A. Electroencephalogram (EEG) based prediction of attention deficit hyperactivity disorder (ADHD) using machine learning. *Appl Neuropsychol Adult* 2023**:** 1-12.

211. Altinkaynak M, Dolu N, Guven A, Pektas F, Ozmen S, Demirci E *et al.* Diagnosis of Attention Deficit Hyperactivity Disorder with combined time and frequency features. *Biocybernetics and Biomedical Engineering* 2020; **40**(3)**:** 927-937.

212. Altun S, Alkan A, Altun H. Application of deep learning and classical machine learning methods in the diagnosis of attention deficit hyperactivity disorder according to temperament features. *Concurrency and Computation-Practice & Experience* 2022; **34**(13).

213. Altun S, Alkan A, Altun H. Automatic Diagnosis of Attention Deficit Hyperactivity Disorder with Continuous Wavelet Transform and Convolutional Neural Network. *Clin Psychopharmacol Neurosci* 2022; **20**(4)**:** 715-724.

214. Biederman J, Hammerness P, Sadeh B, Peremen Z, Amit A, Or-Ly H *et al.* Diagnostic utility of brain activity flow patterns analysis in attention deficit hyperactivity disorder. *Psychol Med* 2017; **47**(7)**:** 1259-1270.

215. Bledsoe JC, Xiao C, Chaovalitwongse A, Mehta S, Grabowski TJ, Semrud-Clikeman M *et al.* Diagnostic Classification of ADHD Versus Control: Support Vector Machine Classification Using Brief Neuropsychological Assessment. *J Atten Disord* 2020; **24**(11)**:** 1547-1556.

216. Bohland JW, Saperstein S, Pereira F, Rapin J, Grady L. Network, anatomical, and non-imaging measures for the prediction of ADHD diagnosis in individual subjects. *Frontiers in Systems Neuroscience* 2012; (DEC).

217. Brown MRG, Sidhu GS, Greiner R, Asgarian N, Bastani M, Silverstone PH *et al.* ADHD-200 Global Competition: diagnosing ADHD using personal characteristic data can outperform resting state fMRI measurements. *Frontiers in systems neuroscience* 2012; **6:** 69-69.

218. Caye A, Agnew-Blais J, Arseneault L, Goncalves H, Kieling C, Langley K *et al.* A risk calculator to predict adult attention-deficit/hyperactivity disorder: generation and external validation in three birth cohorts and one clinical sample. *Epidemiology and Psychiatric Sciences* 2020; **29**.

219. Cervantes-Henríquez ML, Acosta-López JE, Martinez AF, Arcos-Burgos M, Puentes-Rozo PJ, Vélez JI. Machine Learning Prediction of ADHD Severity: Association and Linkage to ADGRL3, DRD4, and SNAP25. *J Atten Disord* 2022; **26**(4)**:** 587-605.

220. Cervantes-Henriquez ML, Acosta-Lopez JE, Martinez-Banfi ML, Velez JI, Mejia-Segura E, Lozano-Gutierrez SG *et al.* ADHD Endophenotypes in Caribbean Families. *Journal of attention disorders* 2020; **24**(14)**:** 2100-2114.

221. Chaim-Avancini TM, Doshi J, Zanetti MV, Erus G, Silva MA, Duran FLS *et al.* Neurobiological support to the diagnosis of ADHD in stimulant-naïve adults: pattern recognition analyses of MRI data. *Acta Psychiatr Scand* 2017; **136**(6)**:** 623-636.

222. Chen M, Li H, Wang J, Dillman JR, Parikh NA, He L. A Multichannel Deep Neural Network Model Analyzing Multiscale Functional Brain Connectome Data for Attention Deficit Hyperactivity Disorder Detection. *Radiol Artif Intell* 2019; **2**(1)**:** e190012.

223. Chen T, Antoniou G, Adamou M, Tachmazidis I, Su P. Automatic Diagnosis of Attention Deficit Hyperactivity Disorder Using Machine Learning. *Applied Artificial Intelligence* 2021; **35**(9)**:** 657-669.

224. Chen H, Song Y, Li X. A deep learning framework for identifying children with ADHD using an EEG-based brain network. *Neurocomputing* 2019; **356:** 83-96.

225. Cheng W, Ji X, Zhang J, Feng J. Individual classification of ADHD patients by integrating multiscale neuroimaging markers and advanced pattern recognition techniques. *Frontiers in systems neuroscience* 2012; **6:** 58.

226. Chen M, Li H, Fan H, Dillman JR, Wang H, Altaye M *et al.* ConCeptCNN: A novel multi-filter convolutional neural network for the prediction of neurodevelopmental disorders using brain connectome. *Med Phys* 2022; **49**(5)**:** 3171-3184.

227. Chen T, Tachmazidis I, Batsakis S, Adamou M, Papadakis E, Antoniou G. Diagnosing attention-deficit hyperactivity disorder (ADHD) using artificial intelligence: a clinical study in the UK. *Front Psychiatry* 2023; **14:** 1164433.

228. Christiansen H, Chavanon M-L, Hirsch O, Schmidt MH, Meyer C, Mueller A *et al.* Use of machine learning to classify adult ADHD and other conditions based on the Conners' Adult ADHD Rating Scales. *Scientific Reports* 2020; **10**(1).

229. Chu KC, Huang HJ, Huang YS. Validity of Diagnostic Support Model for Attention Deficit Hyperactivity Disorder: A Machine Learning Approach. *J Pers Med* 2023; **13**(11).

230. Chugh N, Aggarwal S, Balyan A. The Hybrid Deep Learning Model for Identification of Attention-Deficit/Hyperactivity Disorder Using EEG. *Clin EEG Neurosci* 2024; **55**(1)**:** 22-33.

231. Crippa A, Salvatore C, Molteni E, Mauri M, Salandi A, Trabattoni S *et al.* The Utility of a Computerized Algorithm Based on a Multi-Domain Profile of Measures for the Diagnosis of Attention Deficit/Hyperactivity Disorder. *Front Psychiatry* 2017; **8:** 189.

232. Dai D, Wang J, Hua J, He H. Classification of ADHD children through multimodal magnetic resonance imaging. *Front Syst Neurosci* 2012; **6:** 63.

233. de Lacy N, Ramshaw MJ, McCauley E, Kerr KF, Kaufman J, Nathan Kutz J. Predicting individual cases of major adolescent psychiatric conditions with artificial intelligence. *Transl Psychiatry* 2023; **13**(1)**:** 314.

234. Deserno MK, Bathelt J, Groenman AP, Geurts HM. Probing the overarching continuum theory: data-driven phenotypic clustering of children with ASD or ADHD. *Eur Child Adolesc Psychiatry* 2022.

235. Dubreuil-Vall L, Ruffini G, Camprodon JA. Deep Learning Convolutional Neural Networks Discriminate Adult ADHD From Healthy Individuals on the Basis of Event-Related Spectral EEG. *Frontiers in neuroscience* 2020; **14:** 251.

236. Duda M, Ma R, Haber N, Wall DP. Use of machine learning for behavioral distinction of autism and ADHD. *Translational Psychiatry* 2016; **6**.

237. Duda M, Haber N, Daniels J, Wall DP. Crowdsourced validation of a machine-learning classification system for autism and ADHD. *Translational Psychiatry* 2017; **7**.

238. Ehrig L, Wagner AC, Wolter H, Correll CU, Geisel O, Konigorski S. FASDetect as a machine learning-based screening app for FASD in youth with ADHD. *NPJ Digit Med* 2023; **6**(1)**:** 130.

239. Eloyan A, Muschelli J, Nebel MB, Liu H, Han F, Zhao T *et al.* Automated diagnoses of attention deficit hyperactive disorder using magnetic resonance imaging. *Frontiers in systems neuroscience* 2012; **6:** 61.

240. Emser TS, Johnston BA, Steele JD, Kooij S, Thorell L, Christiansen H. Assessing ADHD symptoms in children and adults: evaluating the role of objective measures. *Behav Brain Funct* 2018; **14**(1)**:** 11.

241. An Auditory Brainstem Response-Based Expert System for ADHD Diagnosis Using Recurrence Qualification Analysis and Wavelet Support Vector Machine. *Proceedings of the 23rd Iranian Conference on Electrical Engineering*; 2015 May 10-14 2015; Sharif Univ of Technol, Tehran, IRAN2015.

242. Finch HW, Davis A, Dean RS. Identification of individuals with ADHD using the Dean-Woodcock sensory motor battery and a boosted tree algorithm. *Behav Res Methods* 2015; **47**(1)**:** 204-215.

243. Garcia-Argibay M, Zhang-James Y, Cortese S, Lichtenstein P, Larsson H, Faraone SV. Predicting childhood and adolescent attention-deficit/hyperactivity disorder onset: a nationwide deep learning approach. *Mol Psychiatry* 2022.

244. Gaus R, Pölsterl S, Greimel E, Schulte-Körne G, Wachinger C. Can we diagnose mental disorders in children? A large-scale assessment of machine learning on structural neuroimaging of 6916 children in the adolescent brain cognitive development study. *JCPP Adv* 2023; **3**(4)**:** e12184.

245. Han D, Fang Y, Luo H. A Predictive Model Offor Attention Deficit Hyperactivity Disorder Based on Clinical Assessment Tools. *Neuropsychiatr Dis Treat* 2020; **16:** 1331-1337.

246. Haque UM, Kabir E, Khanam R. Early detection of paediatric and adolescent obsessive-compulsive, separation anxiety and attention deficit hyperactivity disorder using machine learning algorithms. *Health Inf Sci Syst* 2023; **11**(1)**:** 31.

247. Hart H, Chantiluke K, Cubillo AI, Smith AB, Simmons A, Brammer MJ *et al.* Pattern classification of response inhibition in ADHD: toward the development of neurobiological markers for ADHD. *Human brain mapping* 2014; **35**(7)**:** 3083-3094.

248. Iannaccone R, Hauser TU, Ball J, Brandeis D, Walitza S, Brem S. Classifying adolescent attention-deficit/hyperactivity disorder (ADHD) based on functional and structural imaging. *European child & adolescent psychiatry* 2015; **24**(10)**:** 1279-1289.

249. Itani S, Rossignol M, Lecron F, Fortemps P. Towards interpretable machine learning models for diagnosis aid: A case study on attention deficit/hyperactivity disorder. *PLoS One* 2019; **14**(4)**:** e0215720.

250. Jahanshahloo HR, Shamsi M, Ghasemi E, Kouhi A. Automated and ERP-Based Diagnosis of Attention-Deficit Hyperactivity Disorder in Children. *J Med Signals Sens* 2017; **7**(1)**:** 26-32.

251. Johnston BA, Mwangi B, Matthews K, Coghill D, Konrad K, Steele JD. Brainstem abnormalities in attention deficit hyperactivity disorder support high accuracy individual diagnostic classification. *Human brain mapping* 2014; **35**(10)**:** 5179-5189.

252. Kaur S, Singh S, Arun P, Kaur D, Bajaj M. Phase Space Reconstruction of EEG Signals for Classification of ADHD and Control Adults. *Clin EEG Neurosci* 2020; **51**(2)**:** 102-113.

253. Kautzky A, Vanicek T, Philippe C, Kranz GS, Wadsak W, Mitterhauser M *et al.* Machine learning classification of ADHD and HC by multimodal serotonergic data. *Transl Psychiatry* 2020; **10**(1)**:** 104.

254. Kiiski H, Bennett M, Rueda-Delgado LM, Farina FR, Knight R, Boyle R *et al.* EEG spectral power, but not theta/beta ratio, is a neuromarker for adult ADHD. *The European journal of neuroscience* 2020; **51**(10)**:** 2095-2109.

255. Kim S, Lee HK, Lee K. Can the MMPI Predict Adult ADHD? An Approach Using Machine Learning Methods. *Diagnostics (Basel)* 2021; **11**(6).

256. Koh JEW, Ooi CP, Lim-Ashworth NS, Vicnesh J, Tor HT, Lih OS *et al.* Automated classification of attention deficit hyperactivity disorder and conduct disorder using entropy features with ECG signals. *Comput Biol Med* 2022; **140:** 105120.

257. Kurokami T, Kobayashi H, Nakajima M, Mikami M, Koeda T. Establishment of an objective index for the diagnosis of attention deficit/hyperactivity disorder by the continuous performance test "MOGRAZ". *Brain Dev* 2022; **44**(10)**:** 664-671.

258. Lan Z, Sun Y, Zhao L, Xiao Y, Kuai C, Xue SW. Aberrant Effective Connectivity of the Ventral Putamen in Boys With Attention-Deficit/Hyperactivity Disorder. *Psychiatry Investig* 2021; **18**(8)**:** 763-769.

259. Lee HJ, Cho S, Shin MS. Supporting diagnosis of attention-deficit hyperactive disorder with novelty detection. *Artif Intell Med* 2008; **42**(3)**:** 199-212.

260. Differentiation between Resting-State fMRI data from ADHD and Normal Subjects : Based on Functional Connectivity and Machine Learning. *Proceedings of the International Conference on Fuzzy Theory and Its Applications (iFUZZY)*; 2012 Nov 16-18 2012; Natl Chung Hsing Univ, Taichung, TAIWAN2012.

261. Lindhiem O, Goel M, Shaaban S, Mak KJ, Chikersal P, Feldman J *et al.* Objective Measurement of Hyperactivity Using Mobile Sensing and Machine Learning: Pilot Study. *JMIR Form Res* 2022; **6**(4)**:** e35803.

262. Loh HW, Ooi CP, Oh SL, Barua PD, Tan YR, Molinari F *et al.* Deep neural network technique for automated detection of ADHD and CD using ECG signal. *Comput Methods Programs Biomed* 2023; **241:** 107775.

263. Lohani DC, Rana B. ADHD diagnosis using structural brain MRI and personal characteristic data with machine learning framework. *Psychiatry Res Neuroimaging* 2023; **334:** 111689.

264. Luo Y, Alvarez TL, Halperin JM, Li X. Multimodal neuroimaging-based prediction of adult outcomes in childhood-onset ADHD using ensemble learning techniques. *NeuroImage Clinical* 2020; **26:** 102238.

265. Maniruzzaman M, Shin J, Hasan MAM, Yasumura A. Efficient Feature Selection and Machine Learning Based ADHD Detection Using EEG Signal. *Cmc-Computers Materials & Continua* 2022; **72**(3)**:** 5179-5195.

266. Mao Z, Su Y, Xu G, Wang X, Huang Y, Yue W *et al.* Spatio-temporal deep learning method for ADHD fMRI classification. *Information Sciences* 2019; **499:** 1-11.

267. McNorgan C, Judson C, Handzlik D, Holden JG. Linking ADHD and Behavioral Assessment Through Identification of Shared Diagnostic Task-Based Functional Connections. *Frontiers in physiology* 2020; **11:** 583005.

268. Mikolas P, Vahid A, Bernardoni F, Süß M, Martini J, Beste C *et al.* Training a machine learning classifier to identify ADHD based on real-world clinical data from medical records. *Sci Rep* 2022; **12**(1)**:** 12934.

269. Predicting ADHD Risk from Touch Interaction Data. *Proceedings of the 20th ACM International Conference on Multimodal Interaction (ICMI)*; 2018 Oct 16-20 2018; Boulder, CO2018.

270. Moghaddari M, Lighvan MZ, Danishvar S. Diagnose ADHD disorder in children using convolutional neural network based on continuous mental task EEG. *Comput Methods Programs Biomed* 2020; **197:** 105738.

271. Mooney MA, Neighbor C, Karalunas S, Dieckmann NF, Nikolas M, Nousen E *et al.* Prediction of Attention-Deficit/Hyperactivity Disorder Diagnosis Using Brief, Low-Cost Clinical Measures: A Competitive Model Evaluation. *Clin Psychol Sci* 2023; **11**(3)**:** 458-475.

272. Mueller A, Candrian G, Kropotov JD, Ponomarev VA, Baschera G-M. Classification of ADHD patients on the basis of independent ERP components using a machine learning system. *Nonlinear biomedical physics* 2010; **4 Suppl 1:** S1-S1.

273. Mueller A, Candrian G, Grane VA, Kropotov JD, Ponomarev VA, Baschera G-M. Discriminating between ADHD adults and controls using independent ERP components and a support vector machine: a validation study. *Nonlinear biomedical physics* 2011; **5:** 5-5.

274. Mueller A, Vetsch S, Pershin I, Candrian G, Baschera G-M, Kropotov JD *et al.* EEG/ERP-based biomarker/neuroalgorithms in adults with ADHD: Development, reliability, and application in clinical practice. *World Journal of Biological Psychiatry* 2020; **21**(3)**:** 172-182.

275. Muthuraman M, Moliadze V, Boecher L, Siemann J, Freitag CM, Groppa S *et al.* Multimodal alterations of directed connectivity profiles in patients with attention-deficit/hyperactivity disorders. *Scientific reports* 2019; **9**(1)**:** 20028.

276. Mwamba HM, Fourie PR, den Heever DV. PANDAS: Paediatric Attention-Deficit/Hyperactivity Disorder Application Software. *Annu Int Conf IEEE Eng Med Biol Soc* 2019; **2019:** 1444-1447.

277. Öztekin I, Finlayson MA, Graziano PA, Dick AS. Is there any incremental benefit to conducting neuroimaging and neurocognitive assessments in the diagnosis of ADHD in young children? A machine learning investigation. *Dev Cogn Neurosci* 2021; **49:** 100966.

278. Öztekin I, Garic D, Bayat M, Hernandez ML, Finlayson MA, Graziano PA *et al.* Structural and diffusion-weighted brain imaging predictors of attention-deficit/hyperactivity disorder and its symptomology in very young (4- to 7-year-old) children. *Eur J Neurosci* 2022; **56**(12)**:** 6239-6257.

279. Oztoprak H, Toycan M, Alp YK, Arikan O, Dogutepe E, Karakas S. Machine-based classification of ADHD and nonADHD participants using time/frequency features of event-related neuroelectric activity. *Clinical Neurophysiology* 2017; **128**(12)**:** 2400-2410.

280. Pereda E, García-Torres M, Melián-Batista B, Mañas S, Méndez L, González JJ. The blessing of Dimensionality: Feature Selection outperforms functional connectivity-based feature transformation to classify ADHD subjects from EEG patterns of phase synchronisation. *PLoS One* 2018; **13**(8)**:** e0201660.

281. Saha P, Sarkar D. Characterization and Classification of ADHD Subtypes: An Approach Based on the Nodal Distribution of Eigenvector Centrality and Classification Tree Model. *Child Psychiatry Hum Dev* 2022.

282. Sen B, Borle NC, Greiner R, Brown MRG. A general prediction model for the detection of ADHD and Autism using structural and functional MRI. *PLoS One* 2018; **13**(4)**:** e0194856.

283. Shi Y, Schulte PJ, Hanson AC, Zaccariello MJ, Hu D, Crow S *et al.* Utility of medical record diagnostic codes to ascertain attention-deficit/hyperactivity disorder and learning disabilities in populations of children. *BMC Pediatr* 2020; **20**(1)**:** 510.

284. Silverstein M, Hironaka LK, Feinberg E, Sandler J, Pellicer M, Chen N *et al.* Using Clinical Data to Predict Accurate ADHD Diagnoses Among Urban Children. *Clin Pediatr (Phila)* 2016; **55**(4)**:** 326-332.

285. Slobodin O, Yahav I, Berger I. A Machine-Based Prediction Model of ADHD Using CPT Data. *Frontiers in Human Neuroscience* 2020; **14:** 560021.

286. Sun Y, Zhao L, Lan Z, Jia X-Z, Xue S-W. Differentiating Boys with ADHD from Those with Typical Development Based on Whole-Brain Functional Connections Using a Machine Learning Approach. *Neuropsychiatric Disease and Treatment* 2020; **16:** 691-702.

287. Tachmazidis I, Chen T, Adamou M, Antoniou G. A hybrid AI approach for supporting clinical diagnosis of attention deficit hyperactivity disorder (ADHD) in adults. *Health Inf Sci Syst* 2021; **9**(1)**:** 1.

288. Tenev A, Markovska-Simoska S, Kocarev L, Pop-Jordanov J, Mueller A, Candrian G. Machine learning approach for classification of ADHD adults. *International Journal of Psychophysiology* 2014; **93**(1)**:** 162-166.

289. Wang X-H, Jiao Y, Li L. Identifying individuals with attention deficit hyperactivity disorder based on temporal variability of dynamic functional connectivity. *Scientific reports* 2018; **8**(1)**:** 11789.

290. Yang CM, Shin J, Kim JI, Lim YB, Park SH, Kim BN. Classifying Children with ADHD Based on Prefrontal Functional Near-infrared Spectroscopy Using Machine Learning. *Clin Psychopharmacol Neurosci* 2023; **21**(4)**:** 693-700.

291. Yasumura A, Omori M, Fukuda A, Takahashi J, Yasumura Y, Nakagawa E *et al.* Applied Machine Learning Method to Predict Children With ADHD Using Prefrontal Cortex Activity: A Multicenter Study in Japan. *Journal of attention disorders* 2020; **24**(14)**:** 2012-2020.

292. Yeh S-C, Lin S-Y, Wu EH-K, Zhang K-F, Xiu X, Rizzo A *et al.* A Virtual-Reality System Integrated With Neuro-Behavior Sensing for Attention-Deficit/Hyperactivity Disorder Intelligent Assessment. *Ieee Transactions on Neural Systems and Rehabilitation Engineering* 2020; **28**(9)**:** 1899-1907.

293. Yoo JH, Kim JI, Kim BN, Jeong B. Exploring characteristic features of attention-deficit/hyperactivity disorder: findings from multi-modal MRI and candidate genetic data. *Brain Imaging and Behavior* 2020; **14**(6)**:** 2132-2147.

294. Zhang-James Y, Helminen EC, Liu J, Franke B, Hoogman M, Faraone SV *et al.* Evidence for similar structural brain anomalies in youth and adult attention-deficit/hyperactivity disorder: a machine learning analysis. *Transl Psychiatry* 2021; **11**(1)**:** 82.

295. Zhu P, Pan J, Cai QQ, Zhang F, Peng M, Fan XL *et al.* MicroRNA profile as potential molecular signature for attention deficit hyperactivity disorder in children. *Biomarkers : biochemical indicators of exposure, response, and susceptibility to chemicals* 2022**:** 1-10.

296. Franz AP, Caye A, Lacerda BC, Wagner F, Silveira RC, Procianoy RS *et al.* Development of a risk calculator to predict attention-deficit/hyperactivity disorder in very preterm/very low birth weight newborns. *J Child Psychol Psychiatry* 2022; **63**(8)**:** 929-938.

297. Lavigne JV, Hopkins J, Ballard RJ, Gouze KR, Ariza AJ, Martin CP. A Precision Mental Health Model for Predicting Stability of 4-year-olds' Attention Deficit/Hyperactivity Disorder Symptoms to Age 6 Diagnostic Status. *Acad Pediatr* 2023.

298. Suresh P, Ray B, Duan K, Chen J, Schoenmacker G, Franke B *et al.* Evaluating the Neuroimaging-Genetic Prediction of Symptom Changes in Individuals with ADHD. *Annu Int Conf IEEE Eng Med Biol Soc* 2021; **2021:** 1950-1956.

299. Zhang Y, Sun Y, Yu Z, Sun Y, Chang X, Lu L *et al.* Risk factors and an early prediction model for persistent methamphetamine-related psychiatric symptoms. *Addict Biol* 2020; **25**(1)**:** e12709.

300. Zhang-James Y, Chen Q, Kuja-Halkola R, Lichtenstein P, Larsson H, Faraone SV. Machine-Learning prediction of comorbid substance use disorders in ADHD youth using Swedish registry data. *J Child Psychol Psychiatry* 2020; **61**(12)**:** 1370-1379.

301. Chang J-C, Lin H-Y, Lv J, Tseng W-YI, Gau SS-F. Regional brain volume predicts response to methylphenidate treatment in individuals with ADHD. *BMC psychiatry* 2021; **21**(1)**:** 26.

302. Faraone SV, Gomeni R, Hull JT, Busse GD, Melyan Z, O'Neal W *et al.* Early response to SPN-812 (viloxazine extended-release) can predict efficacy outcome in pediatric subjects with ADHD: a machine learning post-hoc analysis of four randomized clinical trials. *Psychiatry Res* 2021; **296:** 113664.

303. Kim J-W, Sharma V, Ryan ND. Predicting Methylphenidate Response in ADHD Using Machine Learning Approaches. *The international journal of neuropsychopharmacology* 2015; **18**(11)**:** pyv052.

304. Morrow AS, Campos Vega AD, Zhao X, Liriano MM. Leveraging Machine Learning to Identify Predictors of Receiving Psychosocial Treatment for Attention Deficit/Hyperactivity Disorder. *Adm Policy Ment Health* 2020; **47**(5)**:** 680-692.

305. Setyawan J, Yang H, Cheng D, Cai X, Signorovitch J, Xie J *et al.* Developing a Risk Score to Guide Individualized Treatment Selection in Attention Deficit/Hyperactivity Disorder. *Value Health* 2015; **18**(6)**:** 824-831.

306. Wang LJ, Kuo HC, Lee SY, Huang LH, Lin Y, Lin PH *et al.* MicroRNAs serve as prediction and treatment-response biomarkers of attention-deficit/hyperactivity disorder and promote the differentiation of neuronal cells by repressing the apoptosis pathway. *Transl Psychiatry* 2022; **12**(1)**:** 67.

307. Wong HK, Tiffin PA, Chappell MJ, Nichols TE, Welsh PR, Doyle OM *et al.* Personalized Medication Response Prediction for Attention-Deficit Hyperactivity Disorder: Learning in the Model Space vs. Learning in the Data Space. *Front Physiol* 2017; **8:** 199.

308. Bellou V, Belbasis L, Konstantinidis AK, Tzoulaki I, Evangelou E. Prognostic models for outcome prediction in patients with chronic obstructive pulmonary disease: systematic review and critical appraisal. *BMJ* 2019; **367:** l5358.

309. Moons KGM, Wolff RF, Riley RD, Whiting PF, Westwood M, Collins GS *et al.* PROBAST: A Tool to Assess Risk of Bias and Applicability of Prediction Model Studies: Explanation and Elaboration. *Ann Intern Med* 2019; **170**(1)**:** W1-W33.

310. Moons KG, de Groot JA, Bouwmeester W, Vergouwe Y, Mallett S, Altman DG *et al.* Critical appraisal and data extraction for systematic reviews of prediction modelling studies: the CHARMS checklist. *PLoS Med* 2014; **11**(10)**:** e1001744.

311. Wolff RF, Moons KGM, Riley RD, Whiting PF, Westwood M, Collins GS *et al.* PROBAST: A Tool to Assess the Risk of Bias and Applicability of Prediction Model Studies. *Ann Intern Med* 2019; **170**(1)**:** 51-58.
